# Periplasmic Acid Stress Increases Cell Division Asymmetry (Polar Aging) of *Escherichia coli*

**DOI:** 10.1371/journal.pone.0144650

**Published:** 2015-12-29

**Authors:** Michelle W. Clark, Anna M. Yie, Elizabeth K. Eder, Richard G. Dennis, Preston J. Basting, Keith A. Martinez, Brian D. Jones, Joan L. Slonczewski

**Affiliations:** 1 Department of Biology, Kenyon College, Gambier, Ohio, United States of America; 2 Department of Mathematics and Statistics, Kenyon College, Gambier, Ohio, United States of America; University of Illinois at Urbana-Champaign, UNITED STATES

## Abstract

Under certain kinds of cytoplasmic stress, *Escherichia coli* selectively reproduce by distributing the newer cytoplasmic components to new-pole cells while sequestering older, damaged components in cells inheriting the old pole. This phenomenon is termed polar aging or cell division asymmetry. It is unknown whether cell division asymmetry can arise from a periplasmic stress, such as the stress of extracellular acid, which is mediated by the periplasm. We tested the effect of periplasmic acid stress on growth and division of adherent single cells. We tracked individual cell lineages over five or more generations, using fluorescence microscopy with ratiometric pHluorin to measure cytoplasmic pH. Adherent colonies were perfused continually with LBK medium buffered at pH 6.00 or at pH 7.50; the external pH determines periplasmic pH. In each experiment, cell lineages were mapped to correlate division time, pole age and cell generation number. In colonies perfused at pH 6.0, the cells inheriting the oldest pole divided significantly more slowly than the cells inheriting the newest pole. In colonies perfused at pH 7.50 (near or above cytoplasmic pH), no significant cell division asymmetry was observed. Under both conditions (periplasmic pH 6.0 or pH 7.5) the cells maintained cytoplasmic pH values at 7.2–7.3. No evidence of cytoplasmic protein aggregation was seen. Thus, periplasmic acid stress leads to cell division asymmetry with minimal cytoplasmic stress.

## Introduction

Asymmetry is a much debated property of the bacterial cell [1–8]; see also Table 1. Some bacteria show morphological and functional asymmetry, such as *Caulobacter crescentus* whose cell division yields a stalked cell and a flagellated cell. Others such as *Escherichia coli* show bilateral symmetry and generate daughter cells that appear functionally equivalent. Yet even *E*. *coli* are asymmetric in that each daughter cell inherits an “old pole” (which existed for one or more previous generations) and a “new pole” formed by septation. The old-pole and new-pole cells may show differential division times and reproductive potential, a property termed cell division asymmetry [4, 7, 9]. Under certain conditions, old-pole cells undergo polar aging, defined as an increase in division time and higher rates of cell death over several generations (generally five or more generations are observed). Polar aging also occurs in stalked cells of *Caulobacter crescentus* [2]. Other bacteria such as rhizobia [10] and mycobacteria [11] show polar “rejuvenation” by elongating at alternate poles. In mycobacteria, old-pole and new-pole cells differ in their resistance to various antibiotics.

**Table 1:** Literature on polar aging in *Escherichia coli* colonies.

Author and Contribution	Experimental Design	Culture Conditions	Statistical Methods	Results and Conclusions
**Stewart et al. (2005)** [4]. Polar aging occurs in bacterial cells with apparent morphological symmetry.	Cells expressed YFP under Pl promoter and *lac* repressor, cultured on agarose pad slide. Inheritance of new and old poles was recorded. Determined if pole age affects growth rate and viability.	Cells were inoculated onto a microscope cavity slide sealed with LB-agarose at 30°C. Cells were tracked on a single plane and video along with images were taken using Metamorph software. Tracked 9 generations for 94 microcolonies. No explicit stress condition was applied.	Cell doubling rates at each generation were averaged per cell position in lineage, forming a bifurcating average tree. Old pole and new pole growth rates were compared by pairwise two-tailed T-test. Control datasets were analyzed to test whether pole age and growth rate values show random distribution.	Pole age and growth rate values show nonrandom distribution. Cells inheriting the new pole have an increased growth rate. Dead cells show greater inheritance of old pole. Old pole cells produce less biomass in their offspring than new pole cells.
**Lindner et al. (2008)** [12]. Old poles inherit a greater proportion of protein aggregates. Protein aggregation decreases growth rate independently of old-pole location.	Tracked protein aggregation by chaperone IbpA-YFP, which tags inclusion bodies. Tested the distribution of protein aggregates (inclusion bodies) between poles.	Cells were inoculated onto a microscope cavity slide sealed with LB-agarose at 37°C. Cells were tracked on a single plane; images were taken by Metamorph. Tracked 9 generations for 12 microcolonies. Streptomycin was used as a stress condition.	Growth rates were calculated by exponential fit to cell length increase as a function of time. Growth rates were normalized to the generation means. Old pole and new pole growth rates were compared by t-test for normally distributed, unpaired data. Equal variance was determined by F test.	Young-pole offspring grow faster and old-pole offspring grow more slowly than mother cell. Inclusion bodies (protein aggregates) form at midcell, get stuck in newly formed poles, then stay as pole ages. Inclusion bodies slow growth rates independently of polar location.
**Winkler et al. (2010)** [13]. Heat shock aggregates proteins and increases polar age asymmetry; allows loss of protein aggregates via old-pole cells, and increase of new-cell growth. Nucleoid occlusion (inhibition of septation) drives protein aggregates to the poles.	Cells containing thermolabile proteins linked to YFP under *ara* or *lac* promoter were heat shocked and then placed on a nutrient-dense agarose pad. Cells and protein aggregates were followed as cells divided	Cells were cultured on LB-agarose pad slide at 30°C. During time-lapse experiments, images were captured manually. Growth rates were calculated for each division into two daughter cells. Tracked 4 generations (colonies of 30 cells).	Growth rates were measured for old-pole and new-pole cells cultured continuously for 4 doublings. Mean and standard deviation (SD) were presented.	Under heat stress, old-pole cells consistently inherit polar aggregates. Growth rates decline in aggregate-filled old-pole cells over 4 generations. Cells inheriting new poles with no aggregates show increased growth rates (“rejuvenation”).
**Lele et al. (2011)** [7]. Nutritionally dilute environment selects for cell division symmetry (increased growth yield).	Three strains (113-3D, KL16, 2563) were serially cultured on agar for 2000 generations. Conditions were high glucose (10 mg/ml) or low glucose (0.1 mg/ml).	After 1000 and 2000 generations on agar, growth rate was tested in liquid culture. Cell divisions were observed on agarose pads. Tracked 4–5 generations.	Index of division time asymmetry was calculated using non-parametric Mann Whitney test. Cell lengths were compared by pairwise t-test, one-tailed. Correlation analysis was performed using nonparametric Kendall’s tau test.	Higher glucose concentration increased cell division asymmetry. In all strains, growth yield was negatively correlated with cell division asymmetry. No conditions showed division asymmetry associated with old poles.
**Rang et al. (2012)** [9]. For cells not expressing fluorescent reporters, polar aging (division asymmetry) requires a protein damage agent such as streptomycin.	Strain K-12 MG1655 was cultured by method of Stewart et al. (2005). Inheritance of new and old poles was recorded. Determined if pole age affects growth rate and viability.	Cells were inoculated onto a microscope cavity slide sealed with LB-agarose at 37°C. Conditions included 0,1,2, or 3 μg/ml of streptomycin. Cell growth into microcolonies was captured by time-lapse photography. Tracked 8–9 generations (colonies up to size 400).	Doubling rates of mother and two daughter cells were analyzed with a best-fit linear regression. Three parameters were calculated: the doubling time of the fittest, most damage-free cell; the asymmetry coefficient; and the amount of damage that a cell incurs per unit time.	Cells cultured without streptomycin show no division asymmetry, and zero damage accumulation. The rate of damage accumulation increases with streptomycin concentration (from 0 to 3 μg/ml).
**Lloyd-Price et al. (2012)** [14]. Individual protein aggregates migrate toward a cell pole, with strong bias for the old pole.	Cells expressed a MS2 coat protein fused to a GFP along with a RNA target plasmid. Tracked the migration of individual RNA-MS2-GFP complexes within cells.	Cells were induced for MS2 and cultured on sealed LB-agarose at 37°C supplemented with antibiotics and arabinose inducer. After 1 h, cells were tracked for 2 h with images obtained every minute. Cell division time was 1.5 h (tracked 4 generations).	The degree of biased polar segregation of aggregates was analyzed using the model of biased binomial partitioning of RNA-MS2-GFP complexes.	As cells elongate, RNA-MS2-GFP complexes migrate toward a pole. Complex migration shows strong bias for the old pole.

The fitness advantage of polar aging may derive from partitioning of damage to the cytoplasm [6]. When *Escherichia coli* fissions, each daughter cell inherits cytoplasmic components located at the old poles, as well as components at the mid-cell plane where septation forms the new poles. *E*. *coli* may experience “aging” from the selective partitioning of damaged cytoplasmic components such as denatured and aggregated proteins [12, 13]. The cytoplasmic aggregates are stored preferentially in a pole, and ultimately accumulated by old-pole cells [14]. Thus, cell damage is relegated to an older generation while the younger generation receives the newer components. The asymmetric allocation leads to decreased growth rates and higher mortality rates in the old-pole daughter cells over multiple generations [9, 12, 13, 15]. In effect, the old pole acts as a “disposable soma”, preserving the new pole as an “immortal germ line” [1, 5].

The degree of polar asymmetry depends upon growth conditions. A matrix model of cell growth rate and biomass yield [16] predicts that an environment with high growth potential should favor asymmetry; that is, higher growth rate of new-pole cells (rejuvenation) at the expense of losing the biomass yield of old-pole cells. On the other hand, low nutrient levels and low cytoplasmic stress favor symmetrical cell division [7, 9]. Division asymmetry requires a cytoplasmic damage agent causing protein aggregation, such as streptomycin [9], heat shock [13], or fluorescent reporter proteins [4, 12]. Protein aggregates [12] and individual RNA-MS2-GFP complexes [14] migrate preferentially toward the old pole. For comparison, the experimental design, results, and statistical analysis of key investigations in this field are compiled in Table 1.

Previous reports have focused on the cytoplasm as the source of stress-induced cell division asymmetry. The effects of periplasmic and envelope stress conditions on cell division asymmetry have yet to be tested. An important periplasmic and envelope stress is low pH; for review, see [17, 18]. The periplasm is a major site of acid damage because it equilibrates rapidly with the external environment [19]. *E*. *coli* K-12 growth at pH 4.5–6.0 requires many protective stress responses including amino-acid decarboxylase systems such as the Gad regulon, periplasmic acid chaperones, modulation of outer membrane proteins, and adjustment of proton flux by the electron transport system [17, 20, 21]. Nevertheless, bacteria maintain a high degree of cytoplasmic pH homeostasis. During growth in broth medium over a range of external (periplasmic) pH 6.0–7.5, *E*. *coli* cells maintain cytoplasmic pH at 7.6, in liquid culture [17, 19, 22], or at pH 7.1–7.3, in adherent colonies with perfusion [23]. The cell maintains a remarkably strong cytoplasmic pH homeostasis, despite allowing the periplasmic pH to equal the external pH. Thus, low pH permits a test of primarily periplasmic and envelope stress on cell division asymmetry.

We hypothesized that cells cultured at low extracellular pH would show polar aging or cell division asymmetry, due to periplasmic acid stress. Here, we show that extracellular and periplasmic acid (pH 6.0) increases cell division asymmetry within an *E*. *coli* colony, compared to colonies cultured at pH 7.5. After six generations at external pH 6, the old-pole cell shows consistently longer division time than new-pole cell. This cell division asymmetry occurs despite comparable cytoplasmic pH homeostasis within new-pole and old-pole cells. Cells cultured at pH 7.5, however, show no cell division asymmetry.

## Materials and Methods

### Strains and plasmids

We used JLS1105, a strain of *E*. *coli* K-12 W3110 that contains a pH reporter plasmid, pGFPR01, with pHluorin expression under P_BAD_ [23]. Ratiometric fluorescence of pHlourin was used to measure cytoplasmic pH and observe cell fission. Ampicillin concentration was 50 μg/ml; P_BAD_ promoter was induced with 6 mg/ml L-arabinose.

### Cell preparation and culturing for microscopy

Strain JLS1105 was cultured in LBK (10 g/l tryptone, 5 g/l yeast extract, 10 mM KCl) supplemented with 50 μg/ml ampicillin, 0.2% m/v L-arabinose, and buffered with either 100 mM 3-(N-morpholino)propanesulfonic acid (MOPS) pH 7.5 or 100 mM 2-(N-morpholino)ethanesulfonic acid (MES) pH 5.5 [21, 23]. Cultures were incubated at 37°C with rotation until early stationary phase (10–12 h). Cells were diluted 1:10 in 1-ml aliquots of 0.35% agarose (Amresco, Cat #0710) maintained at 43°C. Agarose-cell suspensions were spread thinly onto a 40-mm round coverslip (Bioptechs) and placed directly into a FCS3 flow cell (Bioptechs) with a chamber volume of 250 μl. The chamber was perfused with LBK medium buffered at pH 6.0 (MES) or at pH 7.5 (MOPS). Medium was warmed to 37°C prior to start of each experiment, and was perfused through the chamber during the observation period as described [23].

### Microscopy

Cells were observed using a 100x oil immersion objective lens on an Olympus BX61WIF-5 microscope. Filters D410 and D470 (Chroma Technology Corp) were used for pHluorin excitation with a xenon arc lamp (LB-LS/ OF17; Sutter Instrument). Fluorescence of adherent cells was observed at excitation wavelengths 410 nm and 470 nm [23]. Fluorescence emission was captured at wavelengths 510–560 nm using filter HQ535. Images were captured and fluorescence intensities were recorded using Metamorph Metafluor software (Molecular Devices) (binning = 1, gain = 0). Wavelength exposure times were controlled for photobleaching. Excitation ratios (470/410) for each fluorescence image within a given experiment were recorded [23]. Adherent cells were located within a field and observed until the first division, so as to confirm viability. After the first successful division, the cells were tracked through at least five more generations of division. Focus was maintained manually, and time-lapse images of the bright field and fluorescent images were acquired at continually decreasing intervals as generation numbers increased. All images were labeled with time stamps and saved in chronological order.

### Image and pole age analysis

Experiments recording at least six generations of growth were analyzed and used to construct cell lineages. During cell fission, each daughter cell inherits an old pole (preexisting in the parent) and a new pole (formed by septation). Each pole retains its initial code throughout the experiment, and each new division introduces a new color code. All phase contrast and fluorescent images within a given experiment were coded manually following a color system that indicated the generation of each pole on every cell as divisions accumulated over time (see lineage for specific color scheme). Fluorescence images captured in the Metafluor program were used concurrently to confirm division.

The coded results were used to create two half-lineages stemming from the first two progeny of the initial parent cell. One half-lineage tracks cell A, and the other tracks cell B. The members of half-lineages A and B are delineated by white dotted lines in each panel with color-coded poles. Within each half-lineage, we identified the cell line retaining the old pole (old-pole cell, numbers 2, 4, 8, 16, 32) as well as the line receiving a new pole at each division (new-pole cell, numbers 3, 6, 13, 26, 53). Division times and inheritance patterns were recorded for all cells within each of 15 half-lineages at external pH 6.0; and for cells within 15 half-lineages at external pH 7.5.

For a given half-lineage to be included in analysis, at least 16 cells (half) had to reach the sixth generation. Lineages include subsequent cell divisions and also indicate the division time of each cell from its initial inception until it divides into two daughter cells. The ages of the two poles (generation number since original cell division) for an individual cell are indicated by two shaded boxes within the lineage. After a given lineage was complete, each cell was assigned a numerical value according to its location within the lineage. Each value was standardized to represent the coordinates of the cell within every half-lineage.

### Statistical analysis

For each tracked colony, mean division times were calculated for the old-pole and new-pole cell lines. Thus, each colony generated paired replicates for half-lineages A and B. In effect, the cell division times were binned over several generations for the line containing the oldest pole and for the line containing the new pole at each division. This binning across generations gave us power to detect relatively subtle differences in division rate.

Over multiple colonies (15 half-lineages at pH 6.0, and another 15 at pH 7.5) the difference between old-pole and new-pole division times was tested for significance using the Wilcoxon signed rank test [24] with modification to include rank data with zeros [25]. Also, an alternative test was conducted, a computational resampling permutation test for paired replicates [26].

### Cytoplasmic pH measurement

Intracellular cytoplasmic measurements were obtained using the pH-dependent ratio between excitation peaks of pHluorin [23]. For the standard curve, the pH gradient was collapsed using 40 mM methylamine hydrochloride and 40 mM potassium benzoate. LBK medium containing 100 mM buffer at pH 4.0–8.5 with a step size of 0.5 of a unit was fitted with a Boltzmann sigmoid best-fit curve, to correlate intracellular 410/470 excitation ratio as a function of pH. The curve is used to set the false-color scale bar to the minimum and maximum values of fluorescence excitation ratio. Typical min and max ratios were 0.45 (pH 4.95) and 1.40 (pH 8.83).

Fluorescence intensities were extracted from raw images acquired in Metafluor. Images were analyzed and plot profiles of intensity levels across individual cells were performed using ImageJ software (http://rsb.info.gov/ij/, created by NIH). Plots of cells in each experimental condition were generated.

## Results

### Cell division asymmetry occurs at external pH 6.0 but not at external pH 7.5

**Fig 1:**
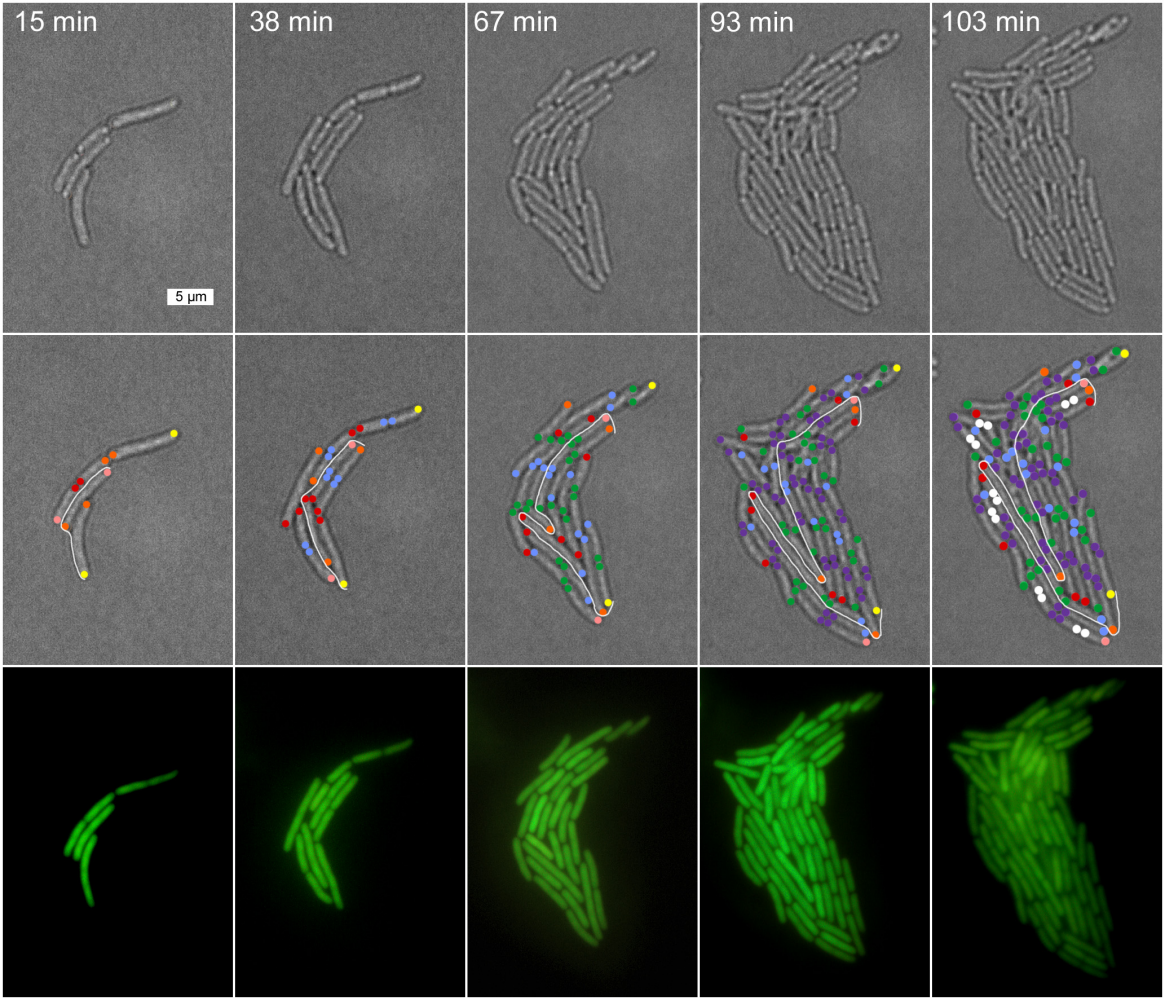
Tracking of individual cells perfused at pH 6.0. The panel above displays time-lapsed images captured during the tracking of cells in a colony for a representative experiment at pH 6.0. Cells are located manually within a field and then followed through six consecutive divisions. The first row of phase contrast images are the original images captured during the experiment. Color coded images below are the phase contrast images marked with relative pole ages (same color scale appearing in lineages). The bottom row of images are corresponding fluorescent images captured at the same time interval as phase contrast images. Scale bar = 5 μm. Time stamps indicate elapsed time from start of the experiment.

**Fig 2:**
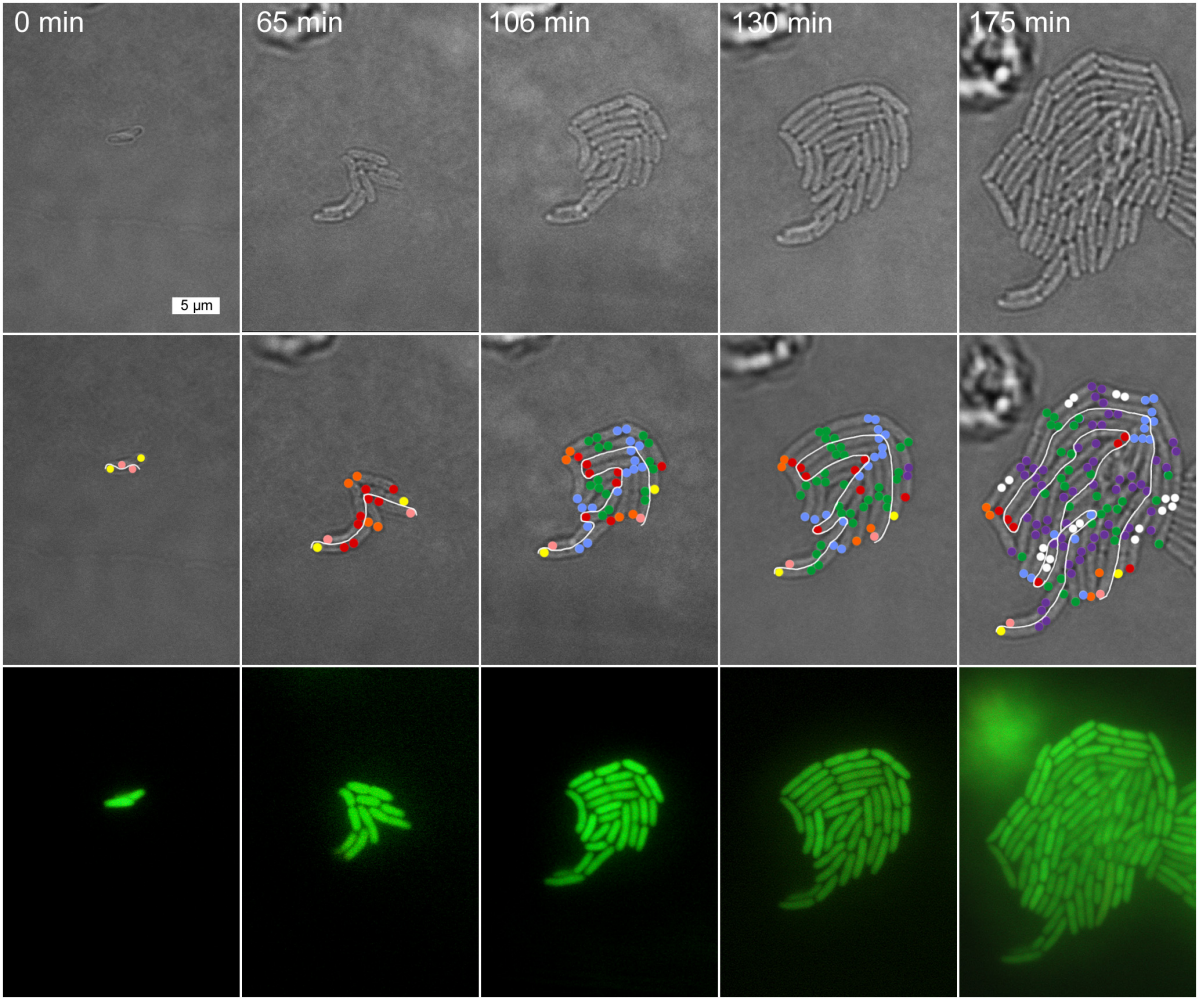
Tracking of individual cells perfused at pH 7.5. The panel above displays time-lapsed images captured during the tracking of cells in a colony for a representative experiment at pH 7.5. Images were obtained and analyzed as in Fig 1.

**Fig 3:**
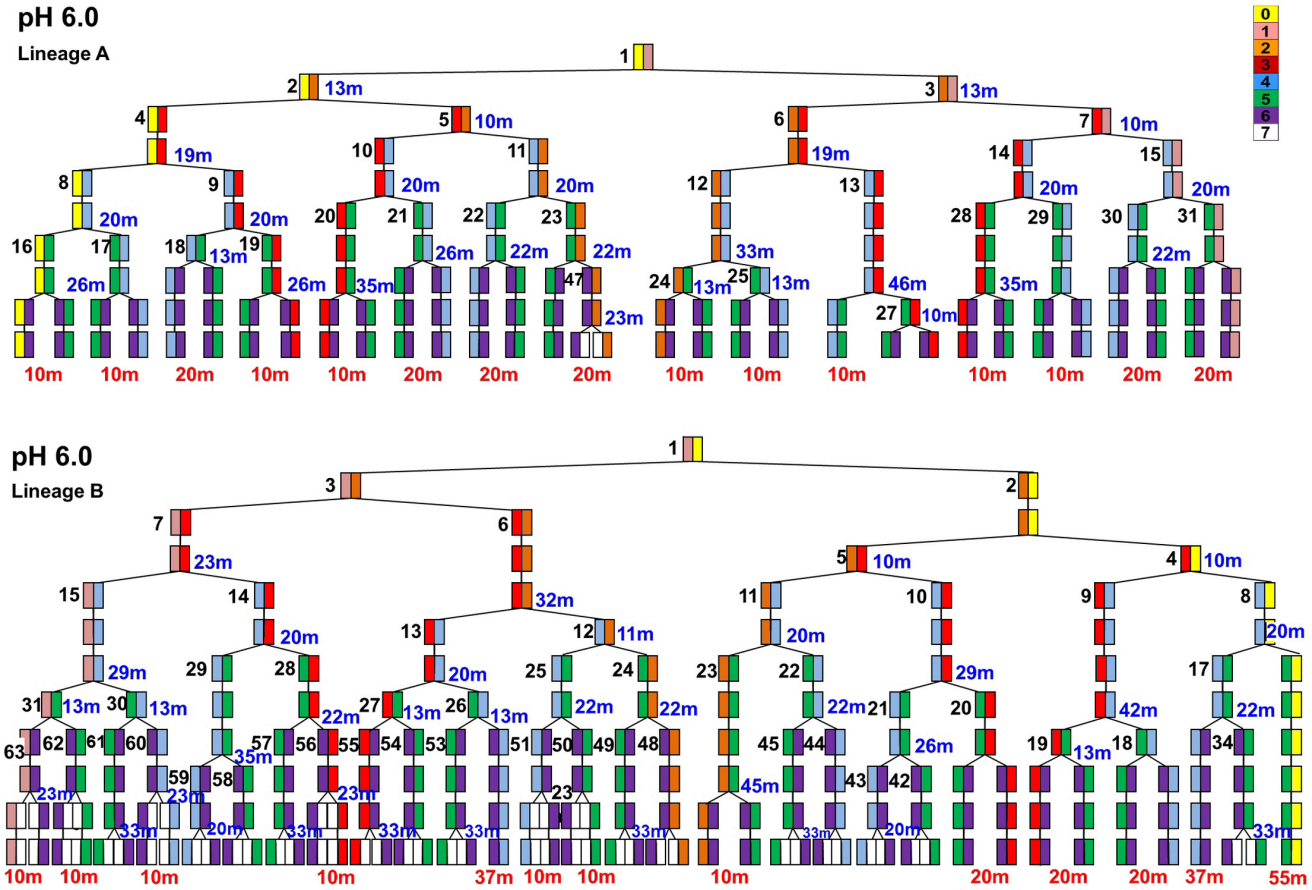
Cell half-lineages A and B in colonies perfused at pH 6.0. Half-lineages A and B each include six generations of cells dividing. Each box represents a single cell at that time point. Individual poles of the cell and their corresponding pole age are labeled according to the color scale in the upper right hand corner. Each cell in the half-lineage is given a distinct number (left of the box). Cell numbers are standardized across all lineages. Colors correspond to the relative polar ages of each cell. Time (min) at each box indicate the division time of that cell, the time from initial existence of the cell until the point where it divided into two daughter cells. Time (min) in red beneath the final division indicates the time the cell existed until the experiment was ended.

**Fig 4:**
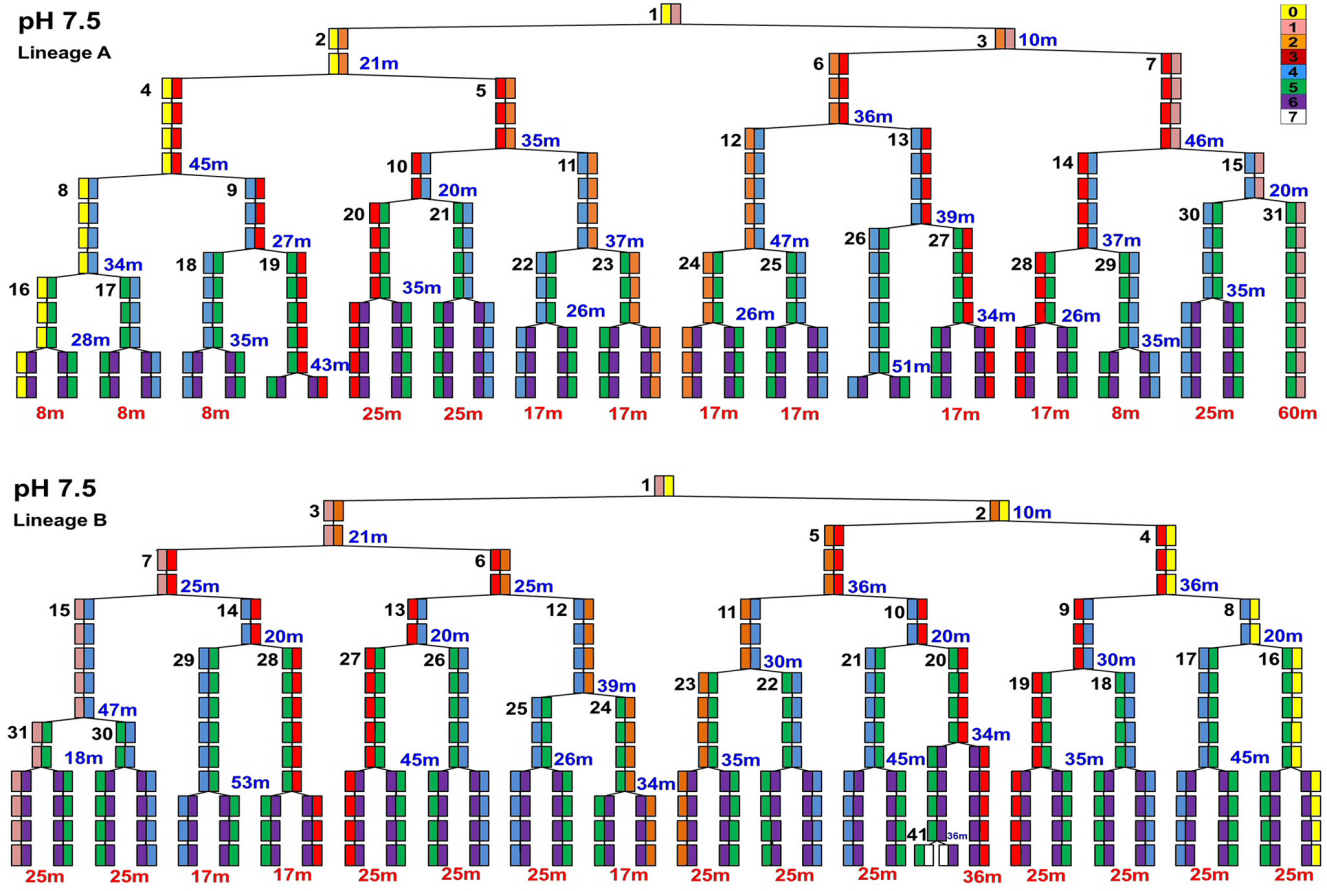
Cell half lineages A and B in colonies perfused at pH 7.5. Half-lineages include six generations of cells dividing. Analysis was conducted as in Fig 3.

Cell division asymmetry was assessed in *E*. *coli* colonies under agarose pads with continual perfusion at pH 6.0 (Fig 1) or at pH 7.5 (Fig 2). The first adherent cell division (generation 0) formed two daughter cells, each with a known old pole and a known new pole. Each of these daughter cells then generated a half-lineage for tracking of old-pole and new-pole cells. Representative pairs of half-lineages are shown in Fig 3 (for external pH 6.0) and in Fig 4 (for external pH 7.0). The half-lineages intertwined with each other within the colony, as shown in Figs 1 and 2. Nevertheless, it was possible to track individually the fate of each cell, as described under Methods. The full set of half-lineages used is shown in **Figs A and B in** S1 File.

Cell division times and inheritance patterns were recorded for 15 half-lineages at external pH 6.0; and for 15 half-lineages at external pH 7.5. Within each half-lineage, we identified the cell line retaining the old pole (old-pole cells, numbered 2, 4, 8, 16, 32). All old-pole cells show a pole color-coded yellow (Figs 3 and 4). We also identified the line of cells receiving a pole that was new at the previous division (new-pole cells, numbered 3, 6, 13, 26, 53). In each case, we compared equivalent cell lines: the line always inheriting the old pole, and the line always inheriting a newly formed pole. Comparison of binned cell division lines revealed cumulative differences in growth rate at low pH, despite the “noise” of individual fluctuations arising from individual cell positions within a colony.

**Fig 5:**
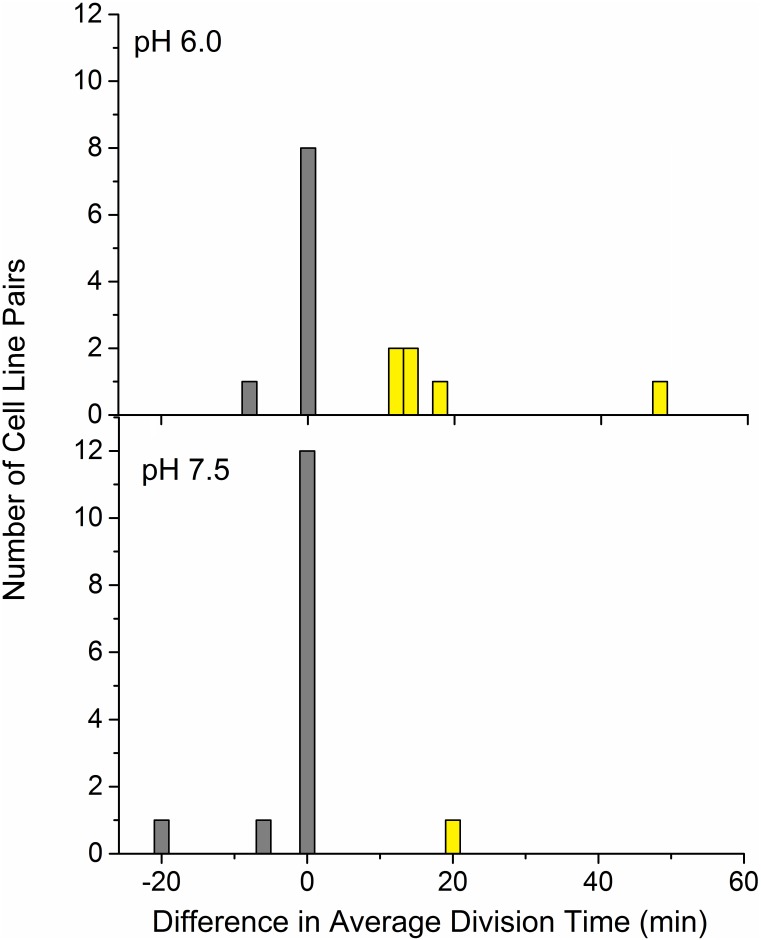
Differences in mean division rates of old-pole and new-pole cell lines. The stacked histograms represent the differences in average division times (old-pole cell line minus new-pole cell line) for each half-lineage. Mean division times for the old-pole line and new-pole line were calculated for each lineage, resulting in replicate pairs. The distribution of these pairwise differences is non-normal (Anderson-Darling p-value < 0.005, indicating strong deviation from normality). Non-parametric tests were used including a Wilcoxon signed rank test and a resampling permutation test.

A histogram for the difference in average division times (old pole line minus new pole line) is plotted in Fig 5. In this histogram, a disproportionate number of cell line pairs with large positive differences in division time would be consistent with a polar aging effect (that is, slowing cell division rates for old-pole cells). The high number of zeros arises with several lineages that have the same total experiment duration time and number of cell divisions, within the resolution of our experiment.

The distribution of the differences of average cell division times was non-normal, requiring a nonparametric (or “distribution-free”) statistical procedures for pairwise comparisons, such as the Wilcoxon signed rank procedure [24]. For lineages of cells perfused at pH 6.0, the Wilcoxon test finds that the old-pole path has a significantly larger mean division time than the new pole path (p = 0.017).

A concern with the standard Wilcoxon test is that it requires reducing the sample by discarding zeros. In rare cases, the large number of zeros can cause the Wilcoxon test to behave inconsistently. For this reason, we also applied a modified Wilcoxon procedure which assures a consistent test by including zeros in the ranking [25]. At pH 6.0, the modified Wilcoxon test found that the old-pole average division time (45.8 ± 4.3 min) exceeds the new-pole average division time (38.1 ± 3.0 min) by 20% (p = 0.017). Thus, the modified Wilcoxon test still found a longer mean division time for the old poles compared to the new poles.

Another nonparametric procedure, a resampling permutation test for paired replicates, uses computer simulation to derive the distribution of test statistics under the assumption of no difference of paired replicate means [26]. In the current study, the permutation test allows the determination of whether a particular sample (i.e. the sample at hand), is inconsistent with the assumption of no difference in mean division times for the old-pole and new-pole paths. After simulating 10,000 resamples, only 149 resamples show a stronger deviation from equal mean division rates than our sample at hand. This result leads to an estimated p-value of 149/10,000 = 0.0149. Like the two previous Wilcoxon tests, the permutation test confirms a larger mean division time for the old-pole cells perfused at pH 6.0.

For cells perfused at pH 7.5, the average mean division rates of the old-pole cells was 45.0 ± 5.3 min, and the average of the new-pole cells was 45.4 ± 4.6 min. The difference (less than 1%) was not found to be significant by any of our tests (p values = 0.789, Wilcoxon; 0.750, modified Wilcoxon; 0.742, permutation test).

### No evidence of cytoplasmic protein aggregation was found

Many studies associate asymmetrical cell divisions with preferential accumulation in the cytoplasm of protein aggregates and inclusion bodies at old poles [12, 13]; see also Table 1. We sought evidence for deposition of aggregates in our tracked colonies. To identify protein aggregation, we quantified the cytosolic fluorescence intensities across the entire length of the cell (**Figs A and B in** S2 File). pHluorin fluorescence should remain diffuse throughout the cytoplasm; any localized sharp increase or decrease in fluorescence values would be an indication of aggregates. Previously, we report observing aggregates or inclusions using pHluorin expressed under a constitutive high-level P_*bsr*_ promoter [23]. Here, we show the same effect (S3 File) in a strain containing the P_*bsr*_ plasmid. Dark regions in the phase-contrast or in the fluorescence micrograph indicate inclusion bodies thought to be composed of misfolded pHluorin.

The arabinose-inducible strain of our present experiment, however, displays normal cell morphology, and never forms detectable inclusion bodies. In the present study, phase contrast and fluorescent intensity profiles were consistent across the entire cell (**Figs A and B in** S2 File). The only variation from the normal curve was a dip in the center of the profile of some cells, indicating septation. There were also slight decreases in fluorescence at the ends of the cell but no sharp peaks or valleys were detected. Thus, while we cannot rule out polar deposition of damaged protein in the cytoplasm, we found no positive evidence of it.

### Cells in tracked colonies maintained cytoplasmic pH homeostasis

In *E*. *coli*, cell growth declines steeply with depression of cytoplasmic pH [27, 28]. We tested whether cytoplasmic pH depression might be involved in pH-dependent polar aging. We used ratiometric pHluorin fluorescence to measure the cytoplasmic pH of all tracked cells. Over the course of the experiment in both conditions, the cytoplasmic pH decreased slightly as cells divided out past five generations. The average cytoplasmic pH of all cells following culture at pH 6.0 was 7.26 ± 0.05, and the average cytoplasmic pH of cells at external pH 7.5 was 7.23 ± 0.13. There was no significant difference between these conditions.

We also compared the cytoplasmic pH of the new-pole cells against old-pole cells within experiments. In colonies perfused at pH 6.0, the average cytoplasmic pH of new-pole cells was 7.28 ± 0.08 and the average pH of the old-pole cells was 7.26 ± 0.08. Although the difference showed significance upon paired t-test (P = 0.035), the cytoplasmic difference of two hundredths of a pH unit was small and unlikely to cause the observed differences in cell division rates. In colonies perfused at pH 7.5, old-pole cells had an average pH of 7.25 ± 0.13 and new-pole cells had an average pH of 7.19 ± 0.18. These values are consistent with previous reports of adherent cell pH at external pH 7.5 [23] and were not significantly different from each other. Thus, both at external pH 6.0 and at pH 7.5, cytoplasmic pH homeostasis was maintained at equivalent levels.

## Discussion

Cell division asymmetry and polar aging have important implications for development of bacterial populations, and for occurrence of antibiotic resistance. Several investigations have examined polar aging of cells under the presence of various external stressors including antibiotics, high temperature, and the overproduction of fluorescent reporters. In these experiments, the occurrence and the mechanism of cell division asymmetry has been controversial. Many researchers find that selective segregation of damaged proteins in these experiments is specific to the stressors used (Table 1), such as streptomycin exposure or protein overexpression [9]. An advantage of our approach is that by binning cell divisions over several generations, we could measure significant effects of an important stress condition (periplasmic acidity) despite concurrent fluctuations in growth patterns within a surface-adherent colony. The significance of our results was confirmed by two different nonparametric tests, the Wilcoxon procedure and the resampling permutation procedure.

The periplasm of *E*.*coli* is strongly affected by acid stress, and its regulation is not well understood. Our work adds new information to polar aging regarding the role of acid stress, at a value that primarily affects the envelope and periplasm [19]. We show that at pH 6, but not pH 7.5 (where periplasmic and cytoplasmic pH are nearly equal), old-pole cells show a longer average generation time and a delay in cell division compared to the new-pole cells. Our results are consistent with the interpretation that asymmetric cell division enables sequestering of damage resulting from periplasmic acid stress. The selective distribution may allow a cell line to exist longer and survive under increasingly toxic conditions [9, 16].

The previous literature points to old-pole accumulation of cytoplasmic protein aggregates as the chief cause of polar aging [12, 13]. While we cannot rule out a cytoplasmic effect during external acid stress, we find no positive evidence for significant cytoplasmic pH stress, nor for protein aggregation (**Figs A and B in** S2 File) in acid-stressed cells. Nevertheless, acid has important effects on the periplasm [29]. The acid-induced protein- folding chaperones HdeA and HdeB protect periplasmic proteins from aggregation and cellular damage [30–32]. It would be interesting to test whether *hdeA* and *hdeB* mutants show increased cell division asymmetry at low external pH.

The outer membrane proteins (OMPs) provide another possible source of pH effect. In the closely related Gram-negative pathogen *Shigella*, the OMPs show polar accumulation in order to modulate host actin-propulsion motility [33]. Thus, we propose that either periplasmic or outer-membrane proteins distribute asymmetrically and may cause polar aging.

## Supporting Information

S1 File Cell half-lineages. Fig A, Cell half-lineages of *E*. *coli* cultured in LBK buffered with 100 mM MES at pH 6.0. The lineages shown were included with those of Fig 2 for experimental analysis of the pH 6.0 condition. All lineages display at least five divisions for a total of at least six generations of cells in each experiment. Individual poles are color coded according to the color bar scale included in the upper right hand corner. Each cell in a half-lineage is also labeled with a distinct number next to the box. Cell numbers are standardized across all lineages. Time (min) at each box indicate the division time of that cell, the time from initial existence of the cell until the point where it divided into two daughter cells. Time (min) in red beneath the final division indicates the time the cell existed until the experiment was ended. Fig B, Cell half-lineages of *E*. *coli* cultured in LBK buffered with 100 mM MOPS at pH 7.5. The lineages shown were included with those of Fig 4 for experimental analysis of the pH 7.5 condition. Cells are labeled as for **Figs A and B in S1 File**.
(PDF)

S2 File Intensity plot profiles of cells. Fig A, Intensity plot profiles of old-pole and new-pole cells at pH 6.0. Cells possessing the oldest pole (A) and the newest pole (B) were scanned end to end, from fluorescent images at 425 nm captured at the end of each experiment. Intensities were quantified using ImageJ (http://rsb.info.gov/ij/). Intensity scale is represented as low (0) to high (300). Lengths of cells are represented as pixels as indicates along the x-axis. Fig B, Intensity plot profiles of old-pole and new-pole cells at pH 7.5. Cells possessing the oldest pole (A) and the newest pole (B) were scanned end to end, and fluorescence was measured as for **Fig A in S2 File**.
(PDF)

S3 File Inclusion bodies observed in E. coli cells expressing pHluorin from P_bsr_. Phase-contrast (top) and ratiometric fluorescence (bottom) images show the strain JLS1013, which expresses pHluorin under the constitutive promoter P_bsr_ [23]. Cultures were incubated at 37°C with rotation to stationary phase (14 h) in LBK media supplemented with 50 μg/ml ampicillin and buffered with 100 mM MOPS at pH 7.5. The cells were suspended in 0.35% agarose and spread on the 40 mm coverslip as described under Methods. The chamber was perfused with LBK media buffered at pH 7.5 (MOPS) during observation. Inclusion bodies resulted in regions of decreased fluorescence (arrow).
(PDF)
