# Supplementary material for: Periplasmic Acid Stress Increases Cell Division Asymmetry (Polar Aging) of Escherichia coli
Source: PLoS One. 2015 Dec 29;10(12):e0144650. doi: 10.1371/journal.pone.0144650 (PMC4694651; doi:10.1371/journal.pone.0144650)

S1 Figure A. Cell Lineages at pH 6.0

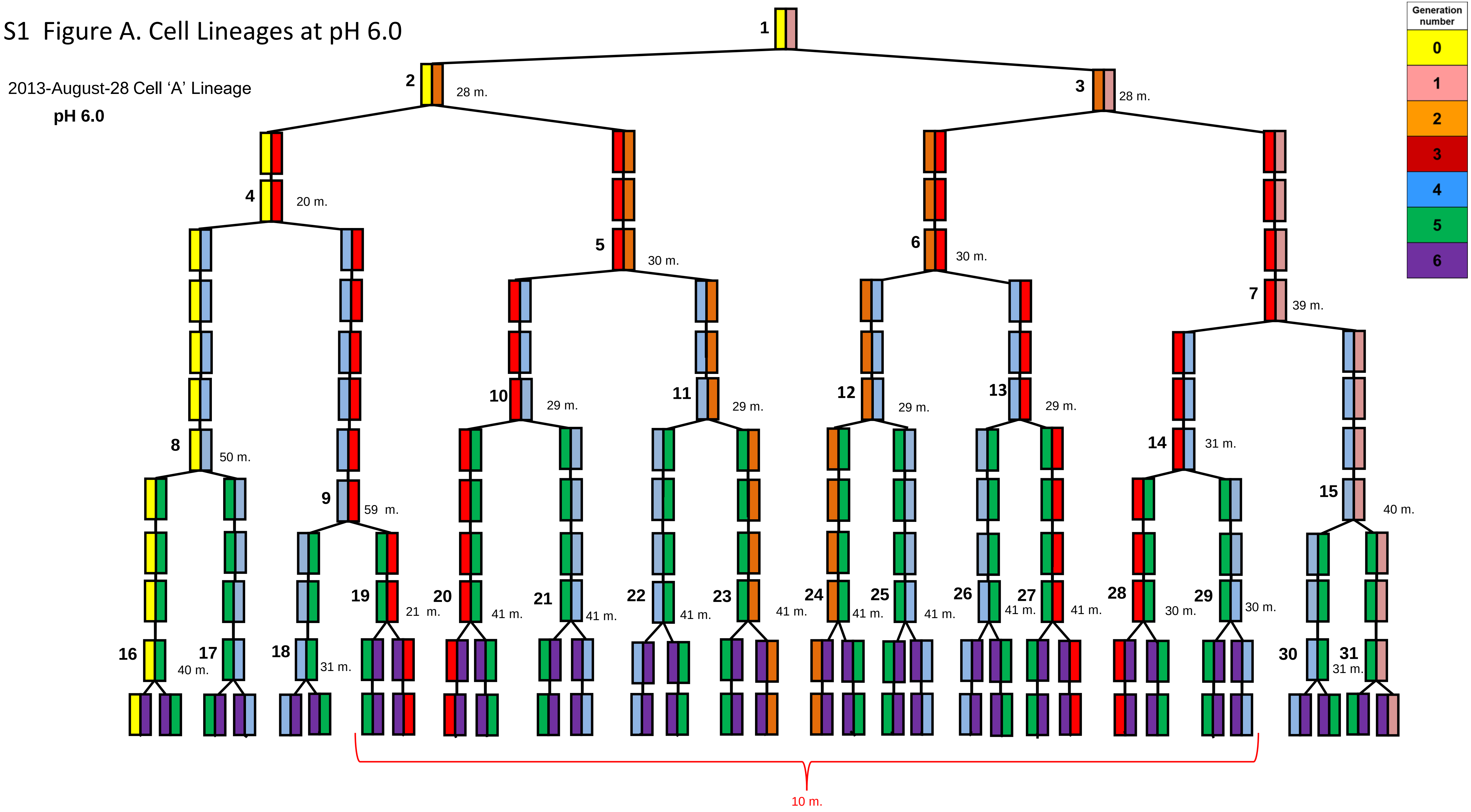

2013-August 28 Cell ‘B’ Lineage

pH 6.0

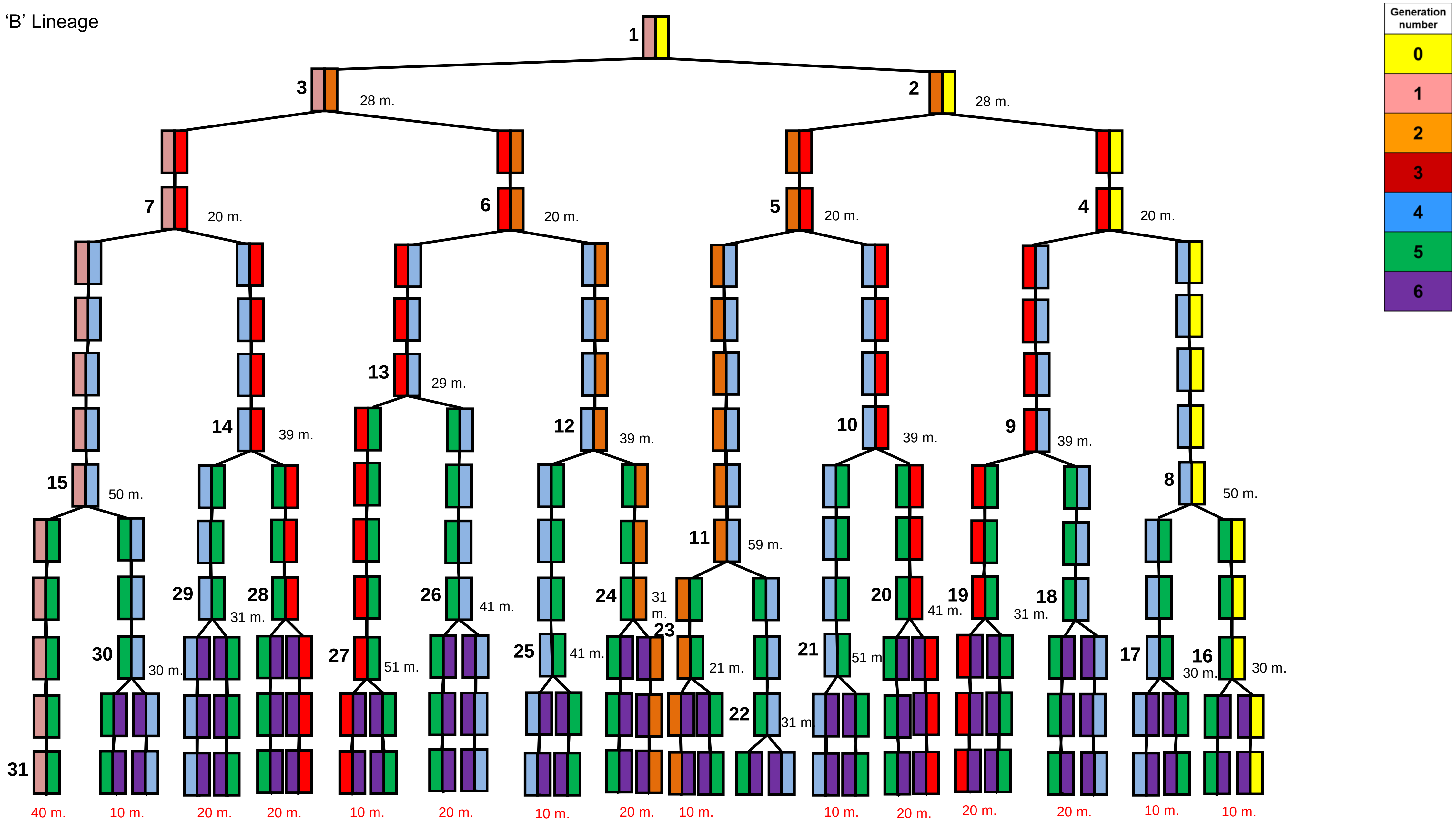

2013-Oct-11 Left Group Cell 'A' Lineage

pH 6.0

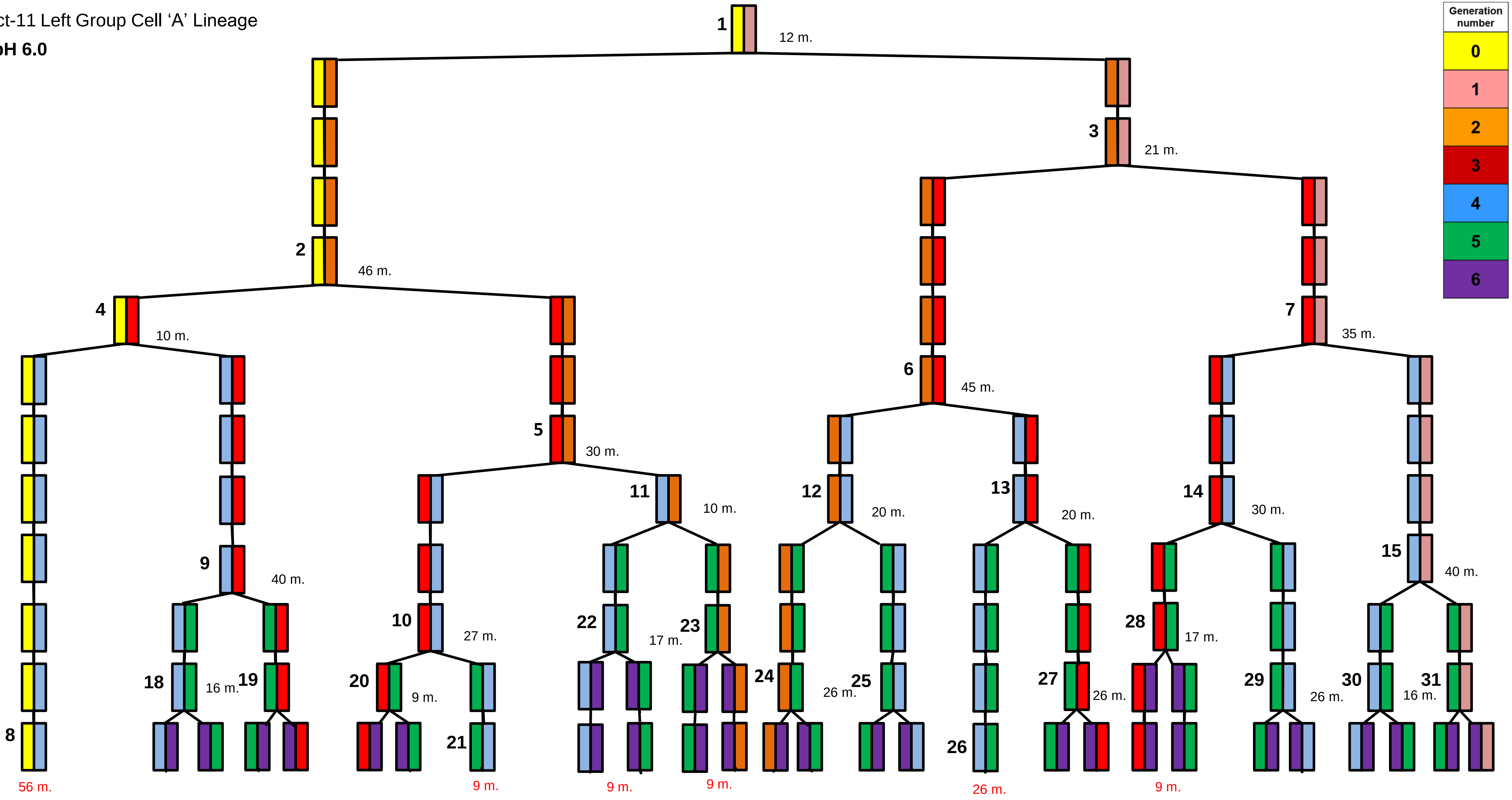

2013-Oct-11 Left Group Cell ‘B’ Lineage (bottom cell)

pH 6.0

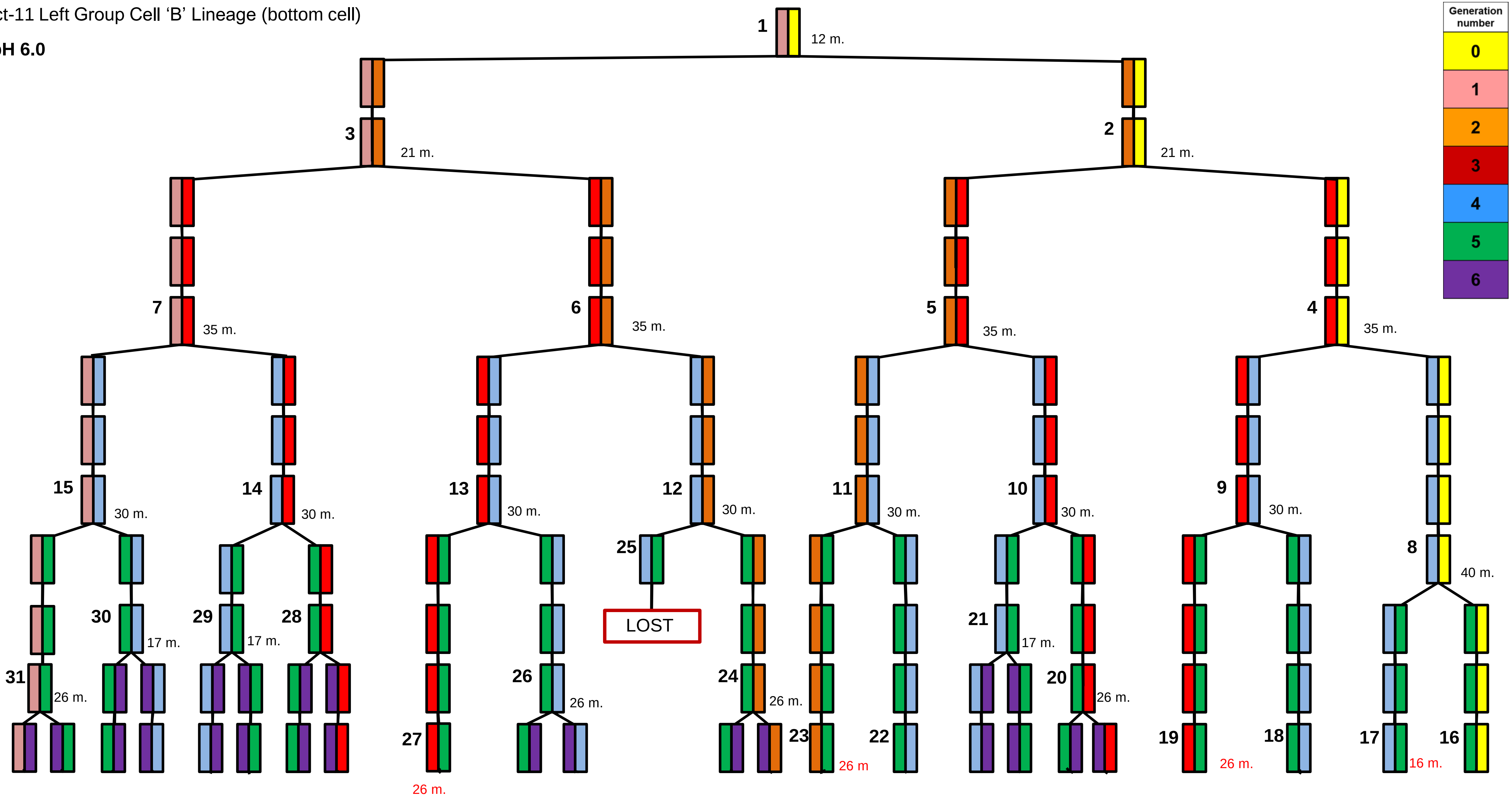

2013-Oct-11 Bottom Group Cell ‘A’ Lineage

pH 6.0

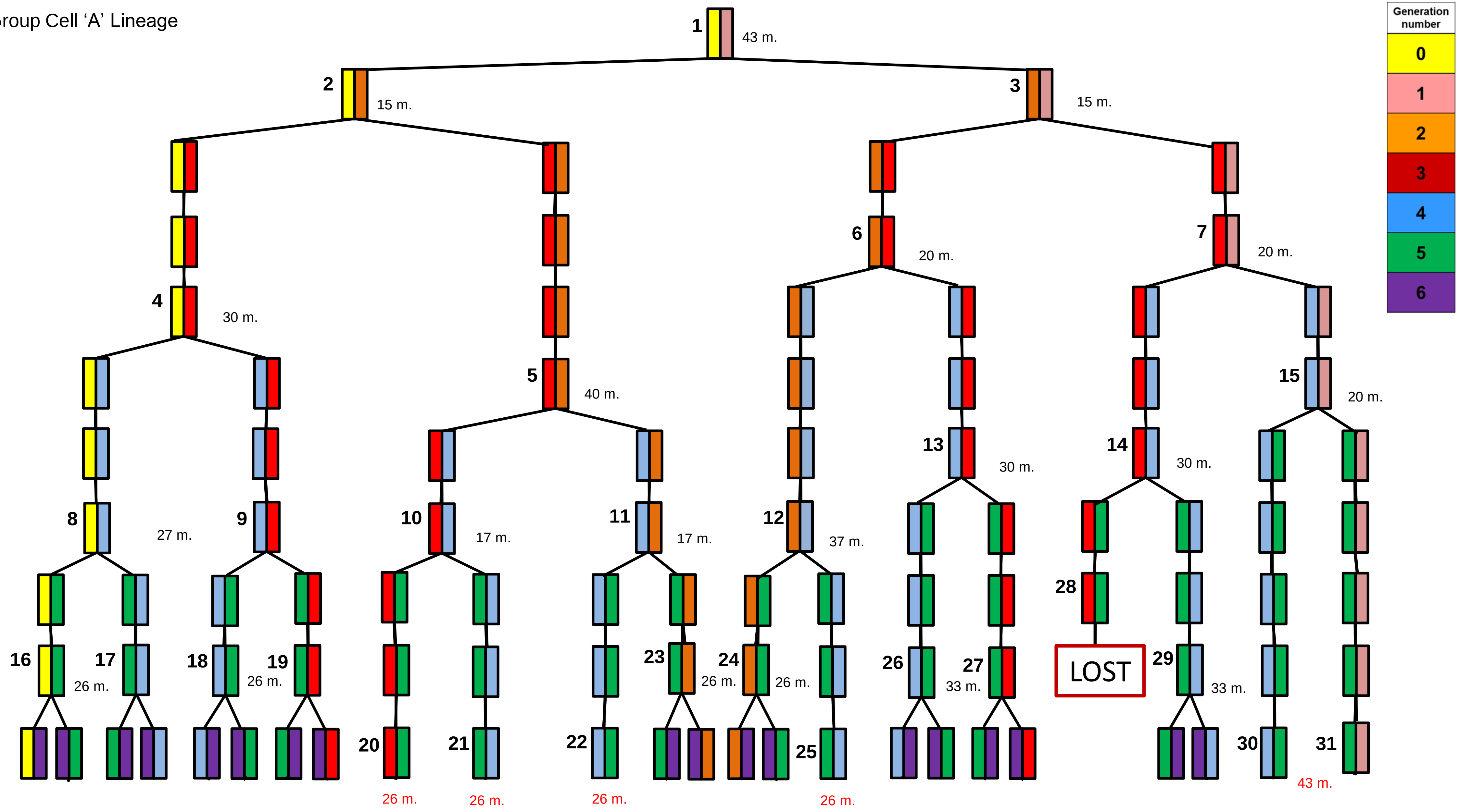

2013-Oct-11 Bottom Group Cell ‘B’ Lineage

pH 6.0

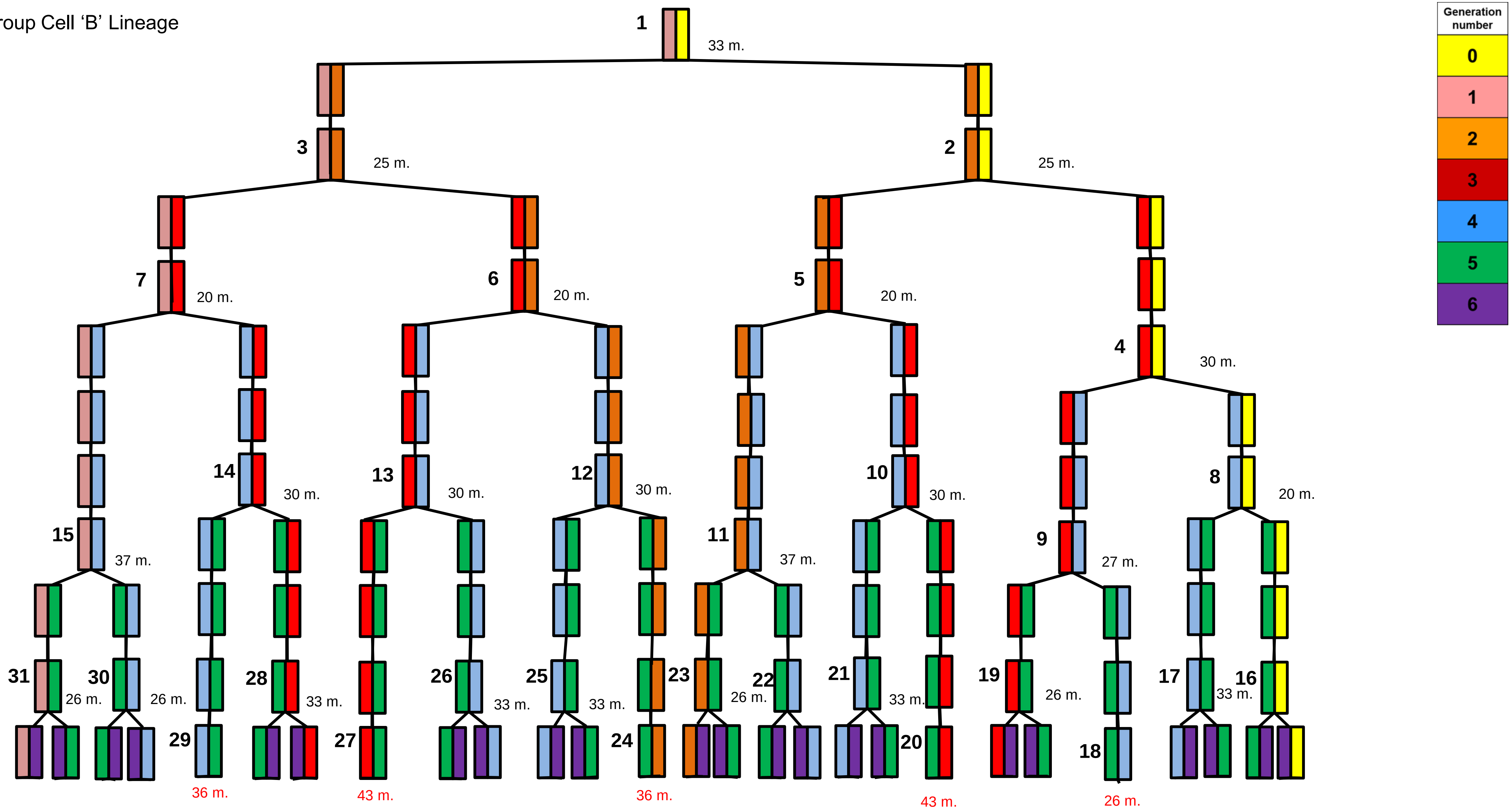

2014-Aug-8 Cell 'A' Lineage

pH 6.0

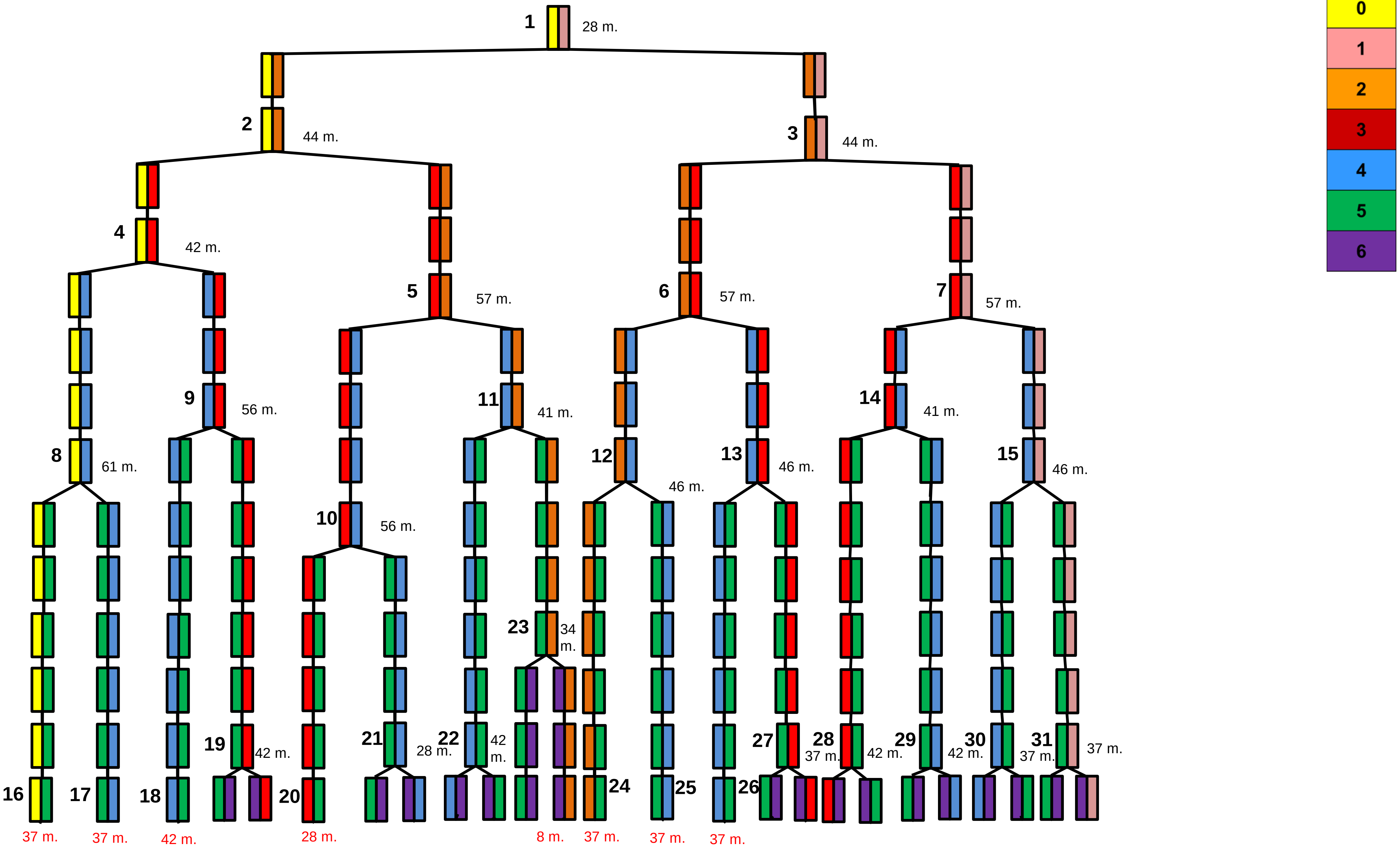

2014-Aug-8 Cell ‘B’ Lineage

pH 6.0

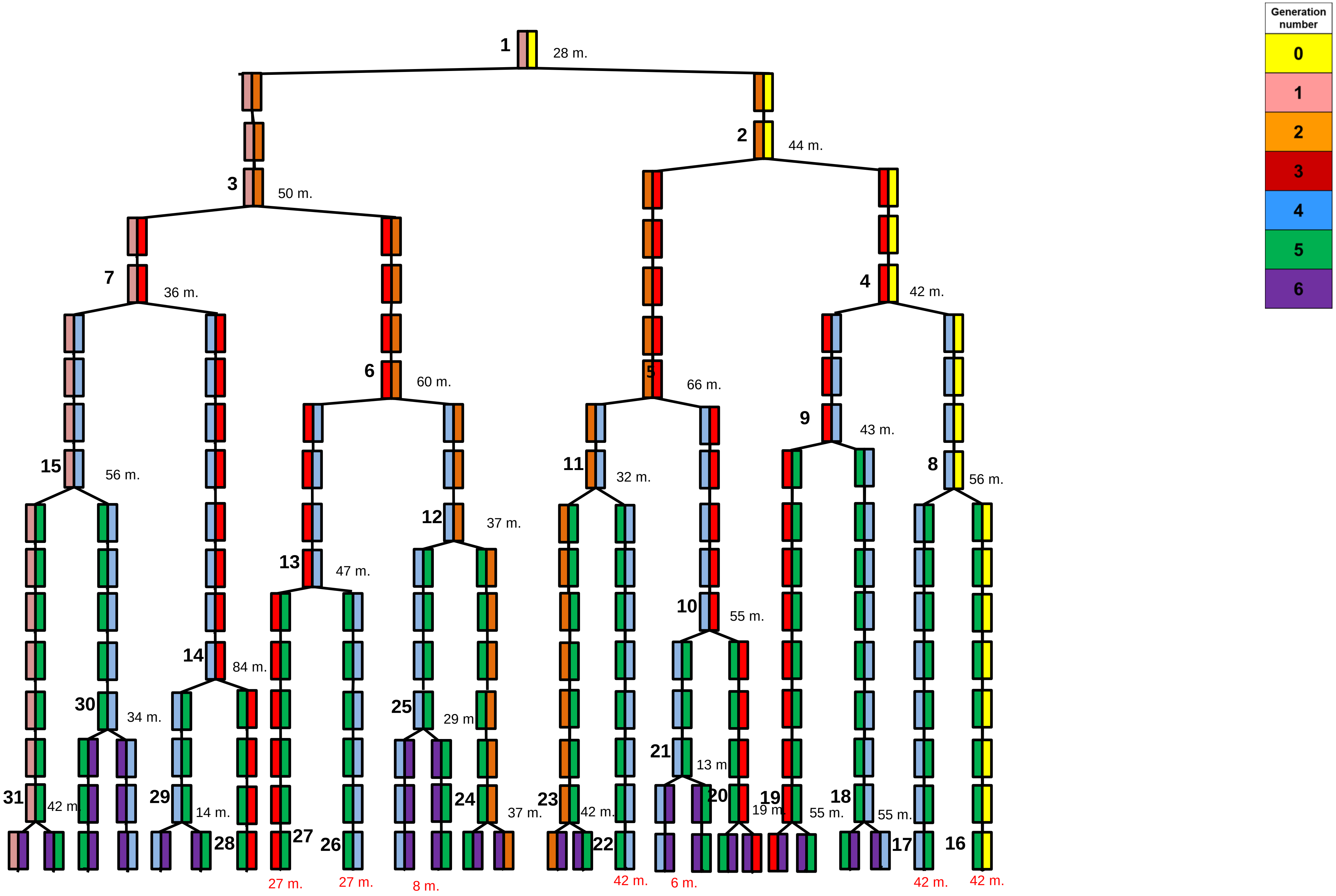

2014-Oct-2 TOP Cell ‘A’

pH 6.0

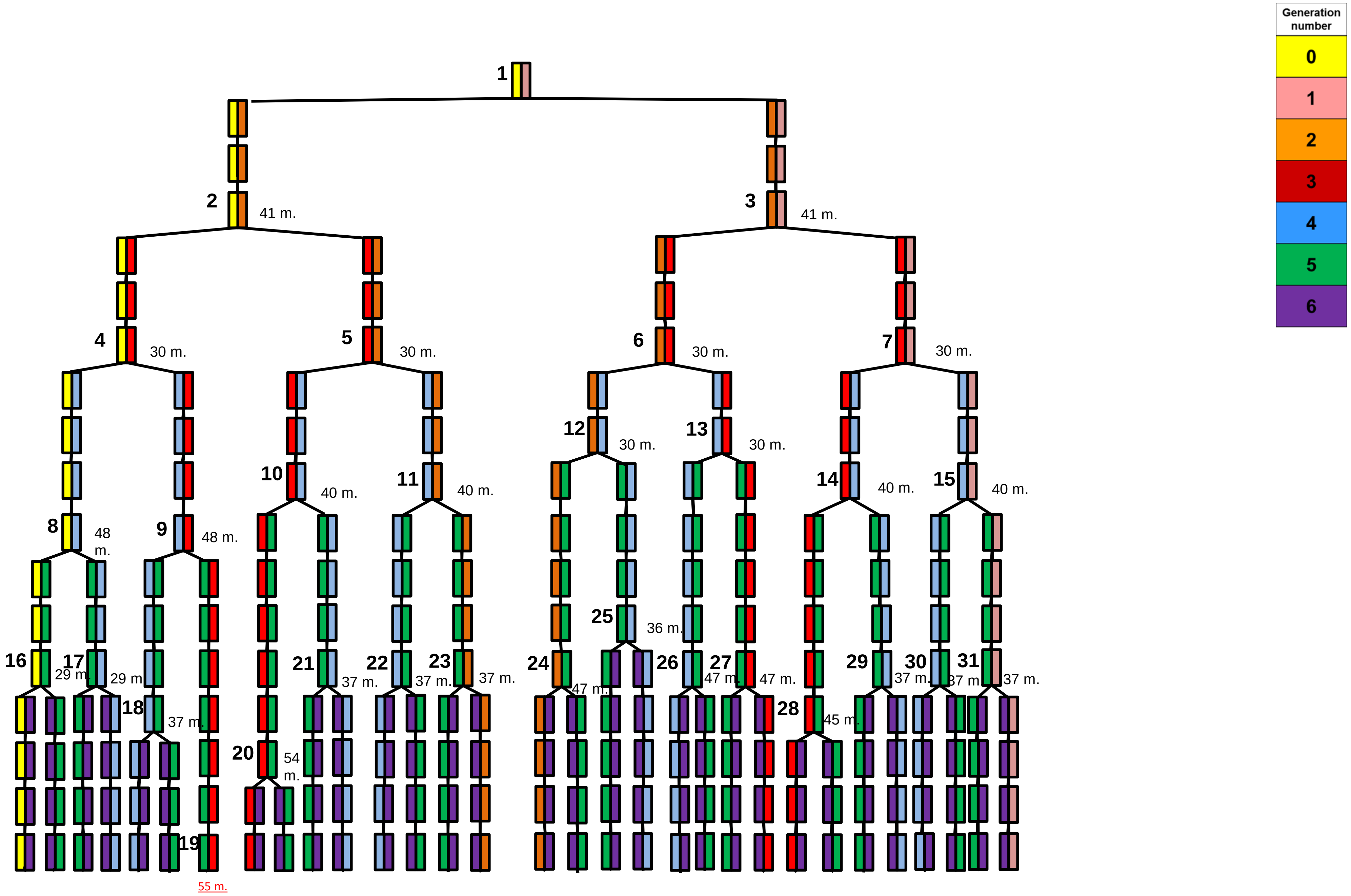

2014-Oct-2 TOP Cell ‘B’

pH 6.0

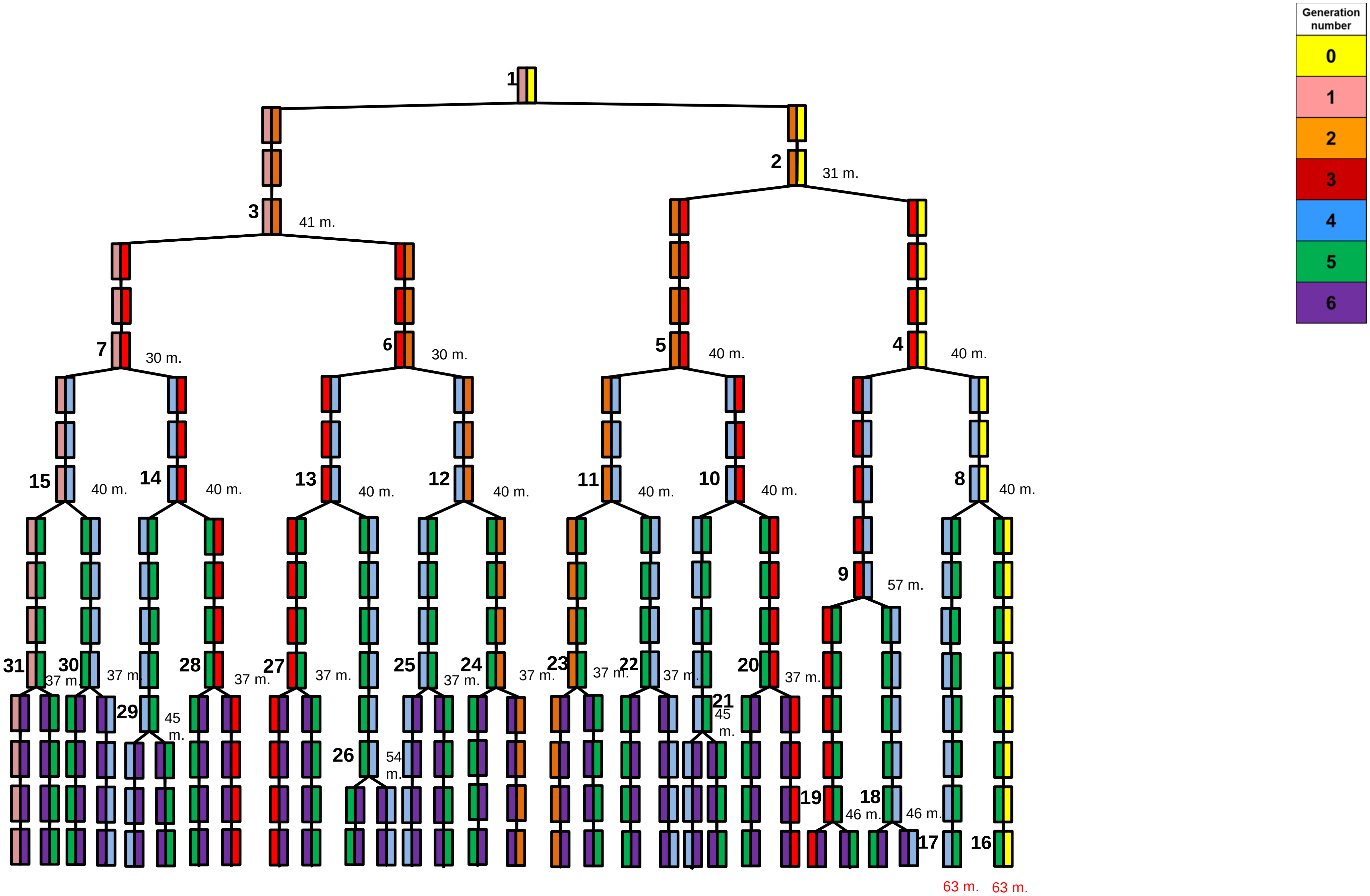

2014-Oct-2 MIDDLE Cell ‘A’

pH 6.0

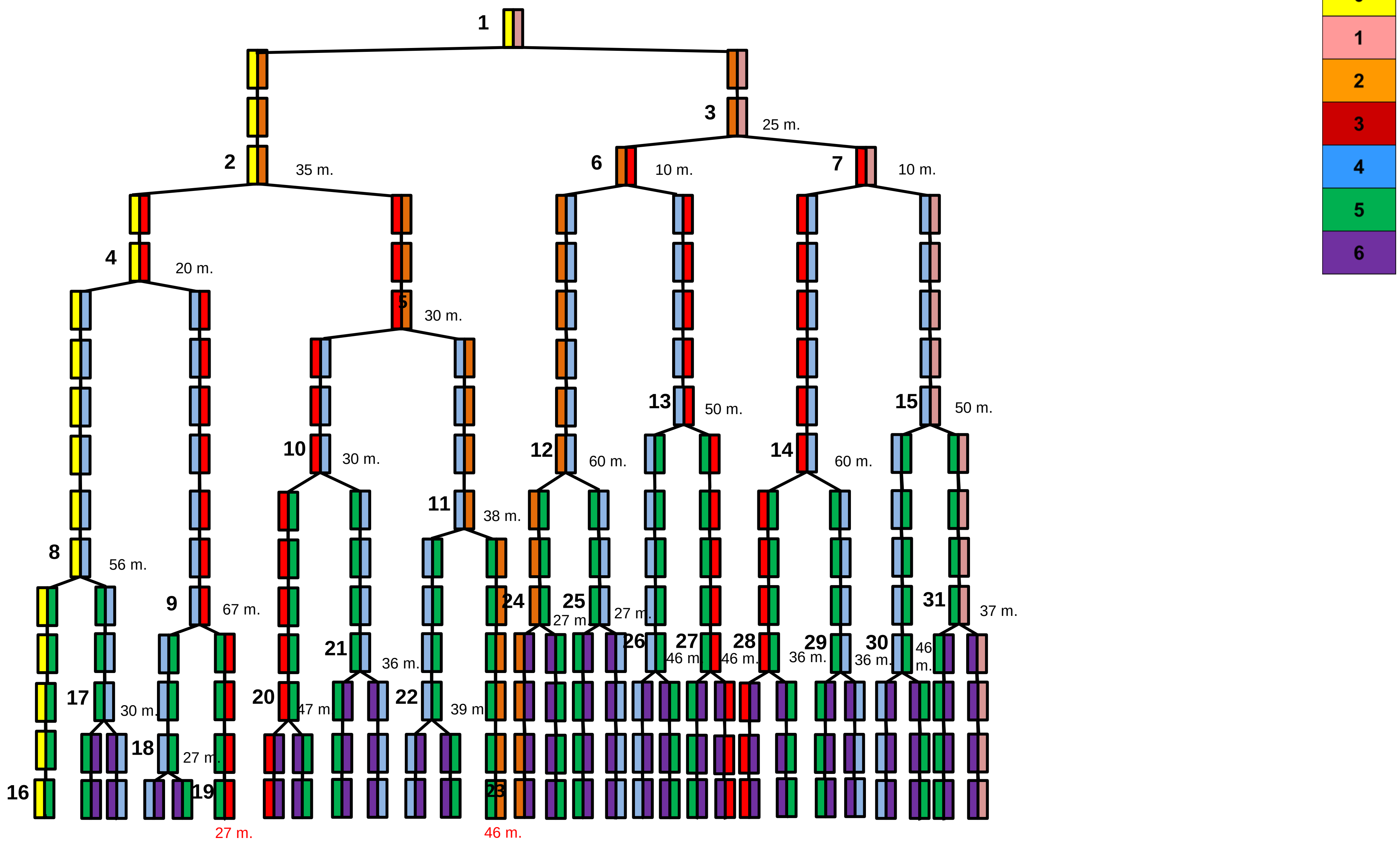

2014-Oct-2 BOTTOM Cell ‘A’

pH 6.0

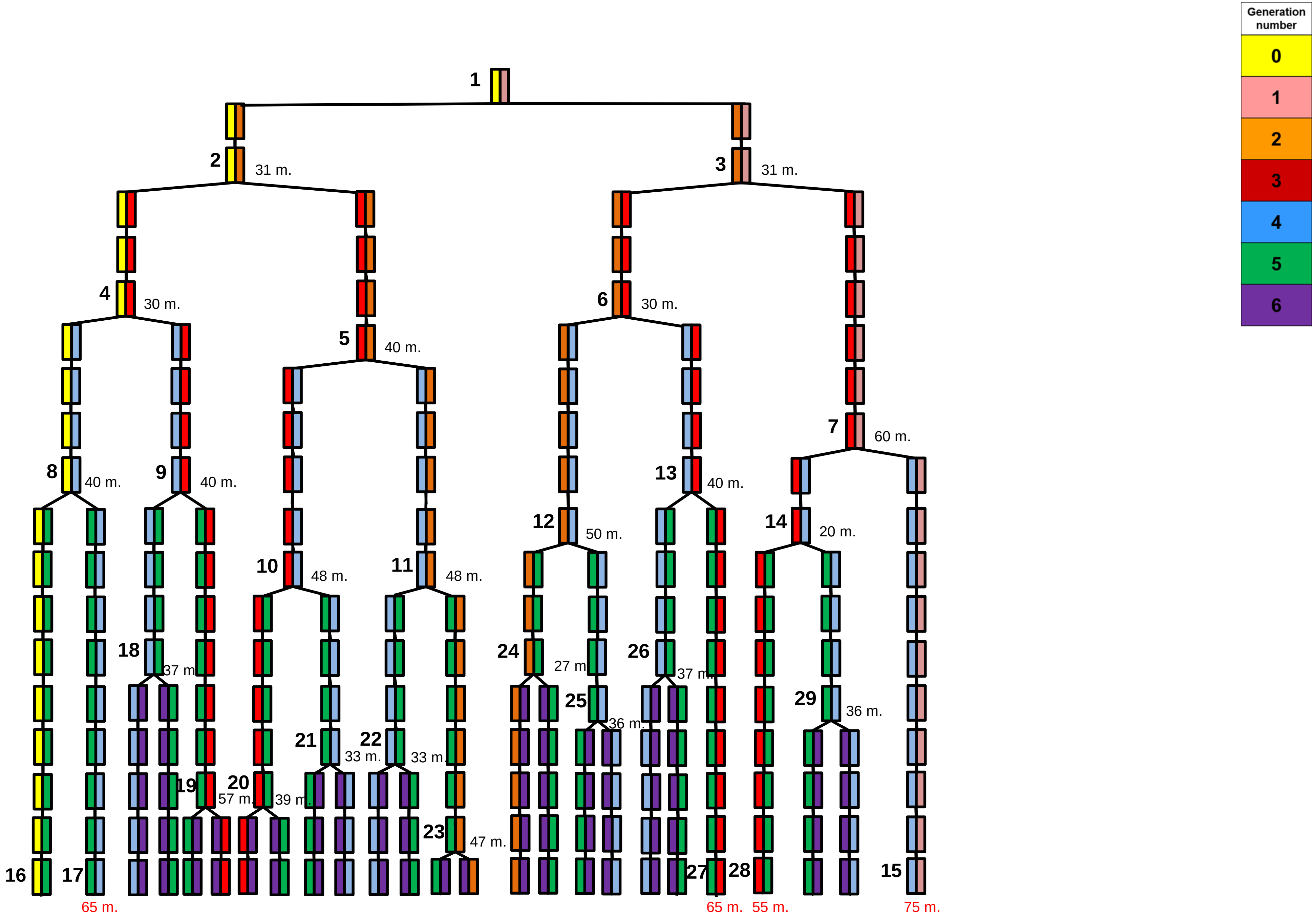

2014-Oct-2 BOTTOM Cell ‘B’

pH 6.0

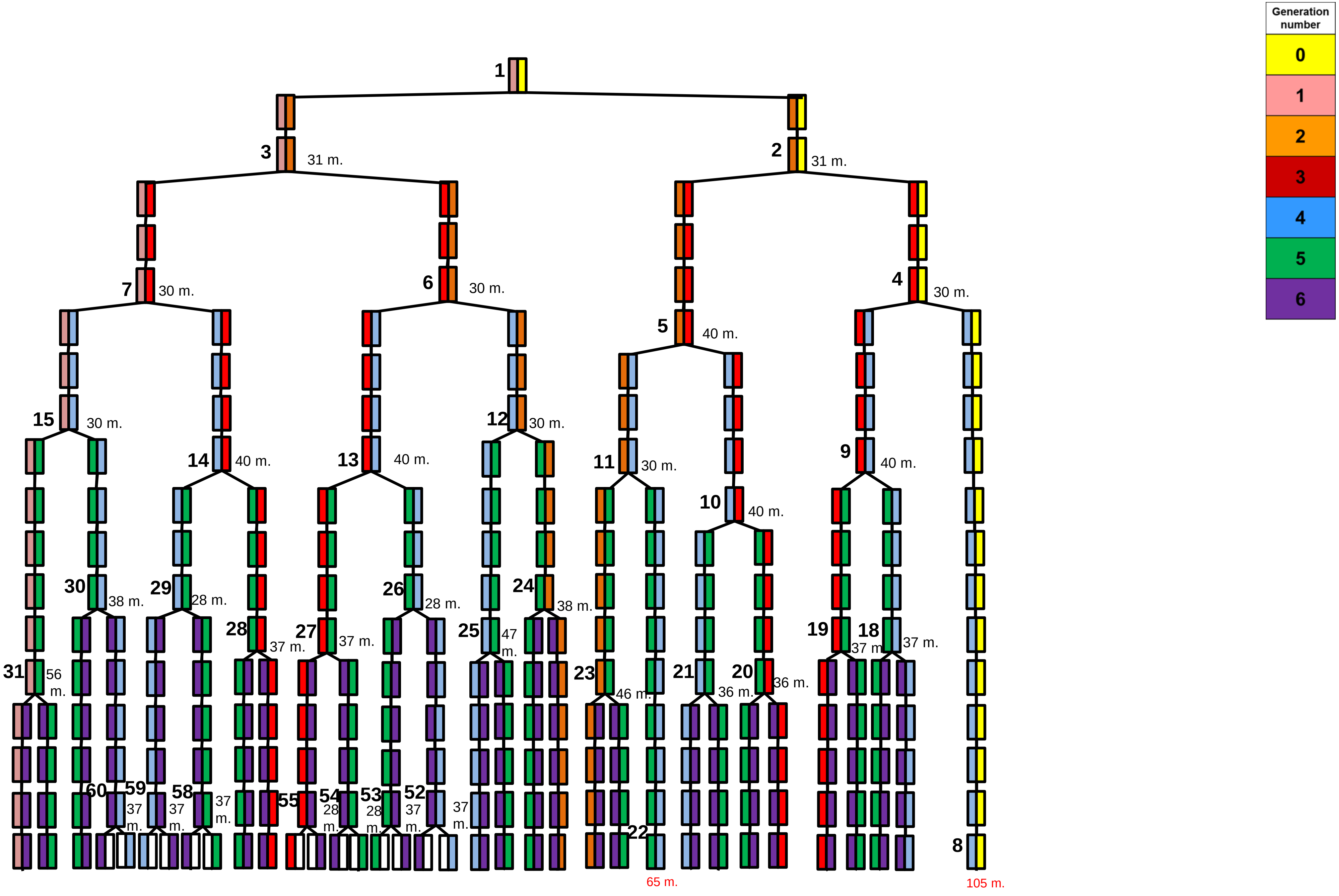

S1 Figure B. Cell Lineages at pH 7.5

2014-Jan-27 Cell ‘A’ Lineage

pH 7.5

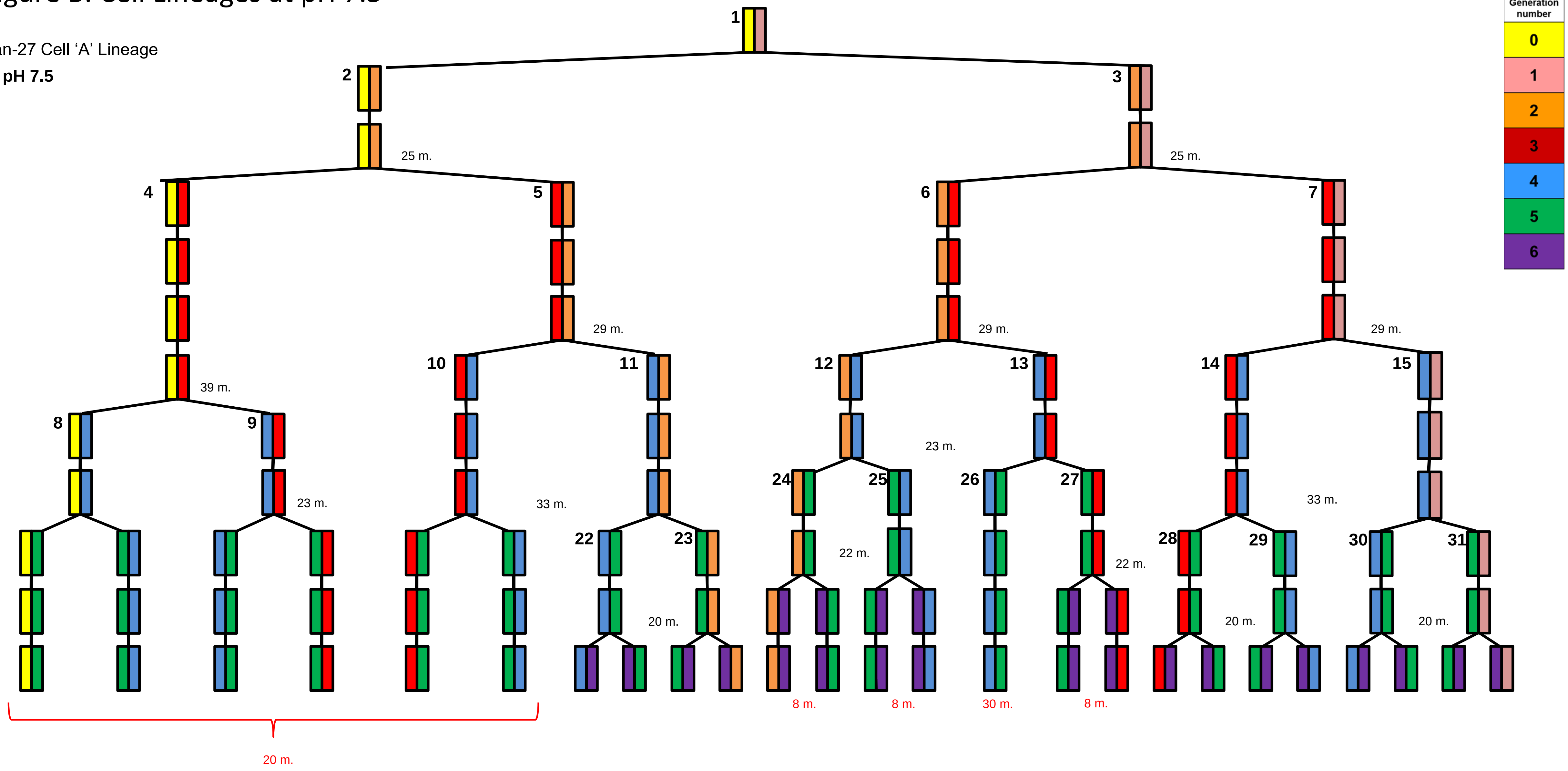

2014-Jan-27 Cell 'B' Lineage

pH 7.5

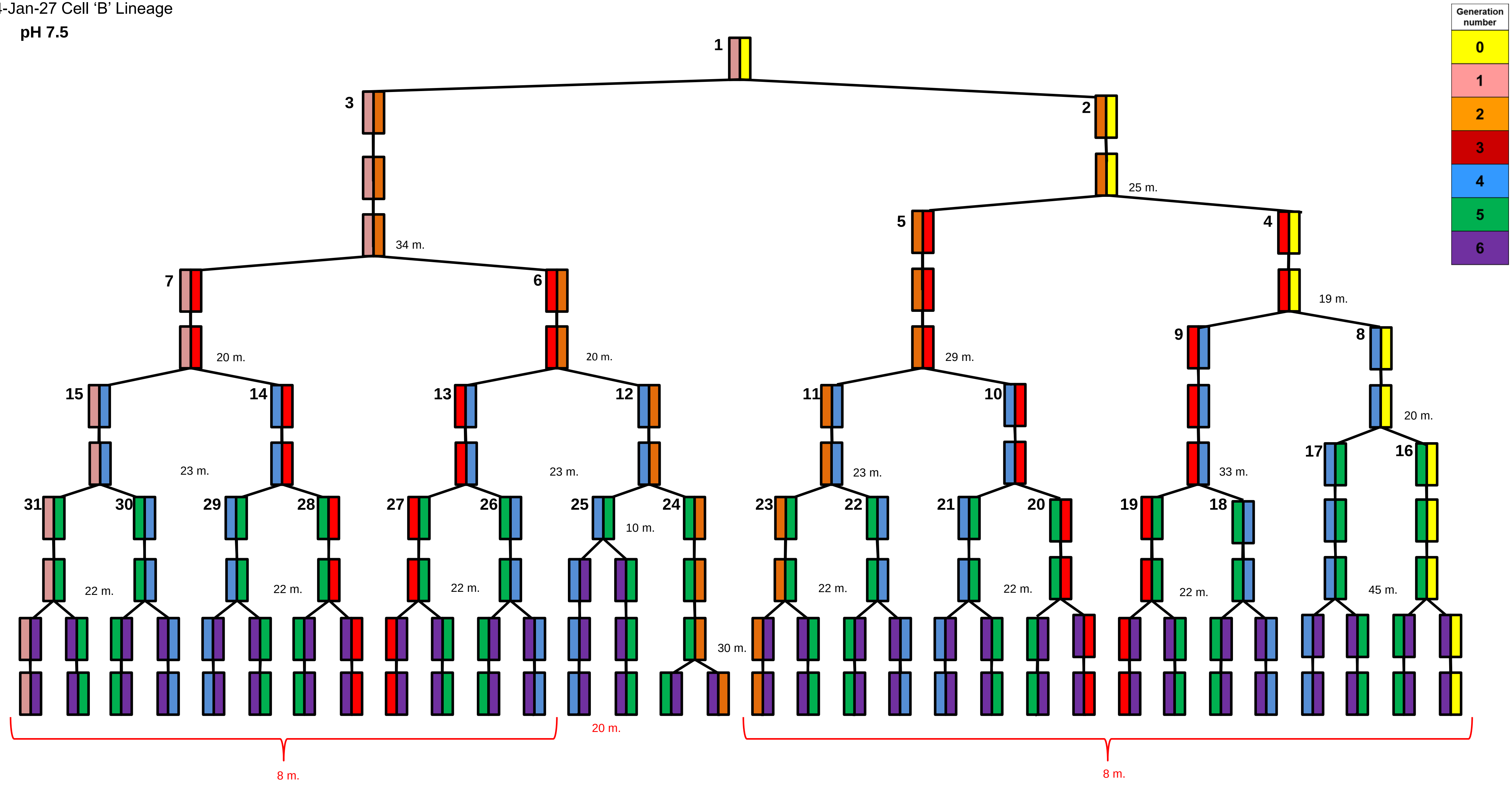

2014-Apr-23 Cell ‘A’ Lineage

pH 7.5

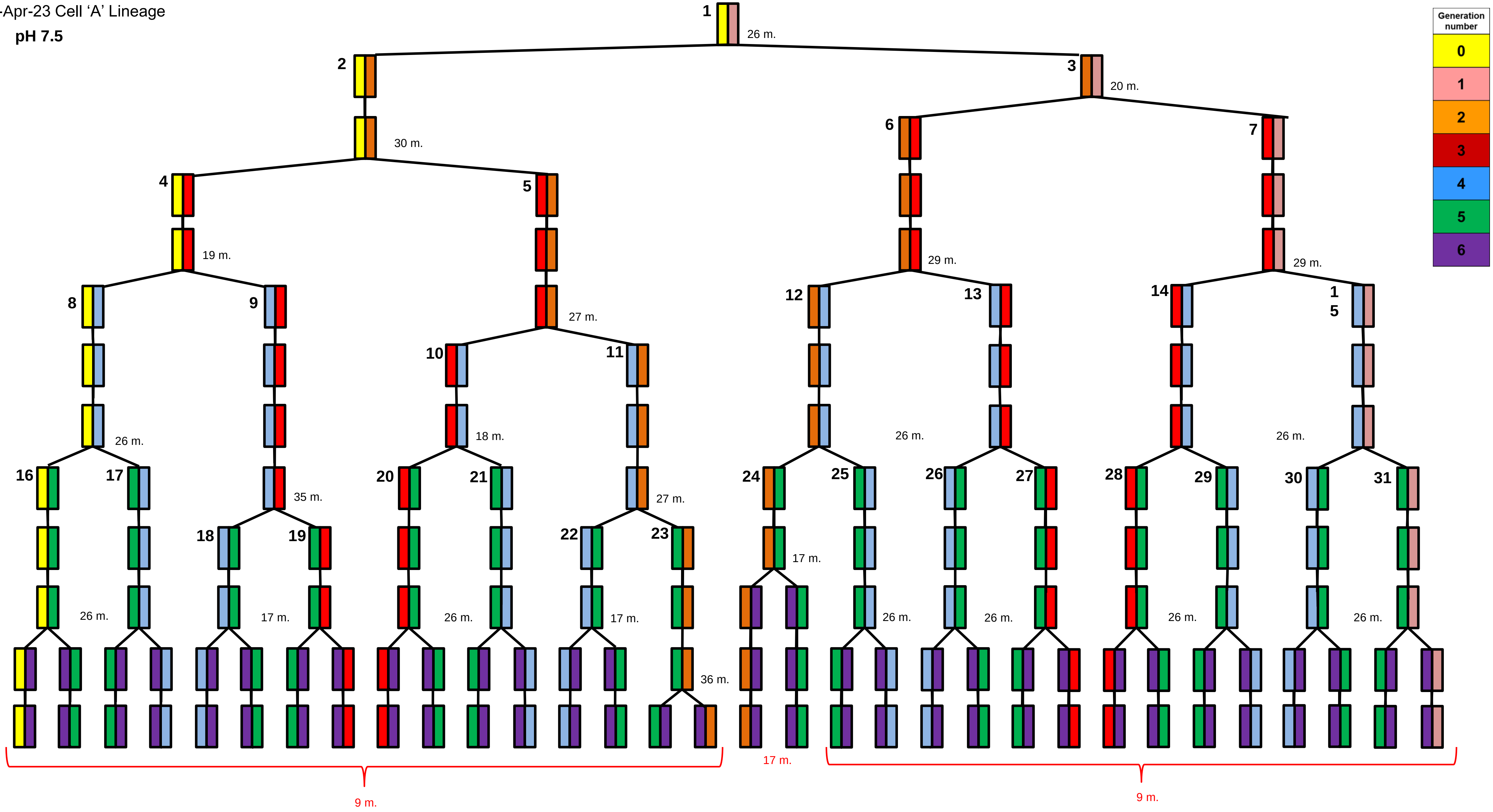

2014-Apr-23 Cell ‘B’ Lineage

pH 7.5

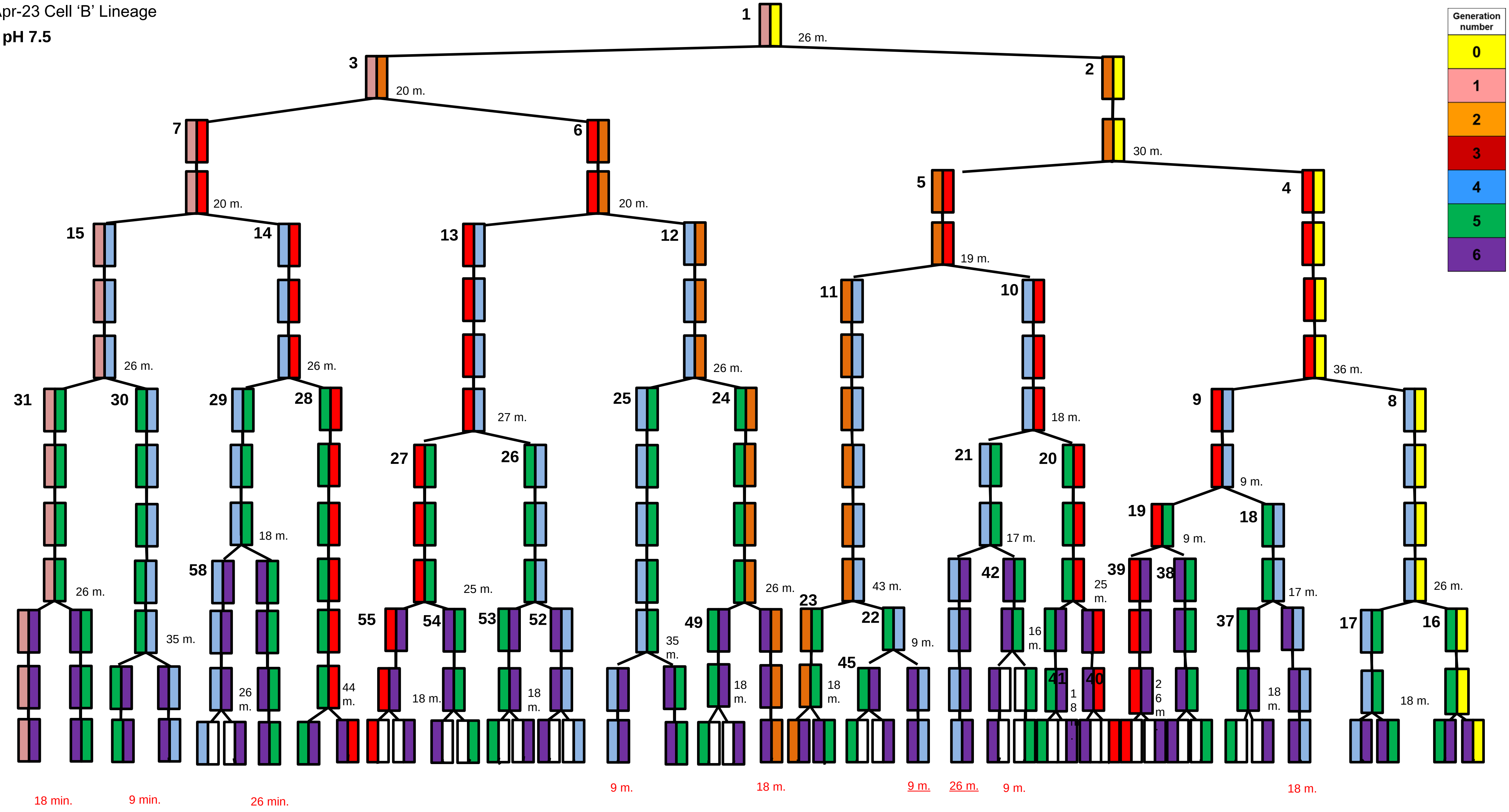

2014-Apr-28 Cell ‘A’ Lineage

pH 7.5

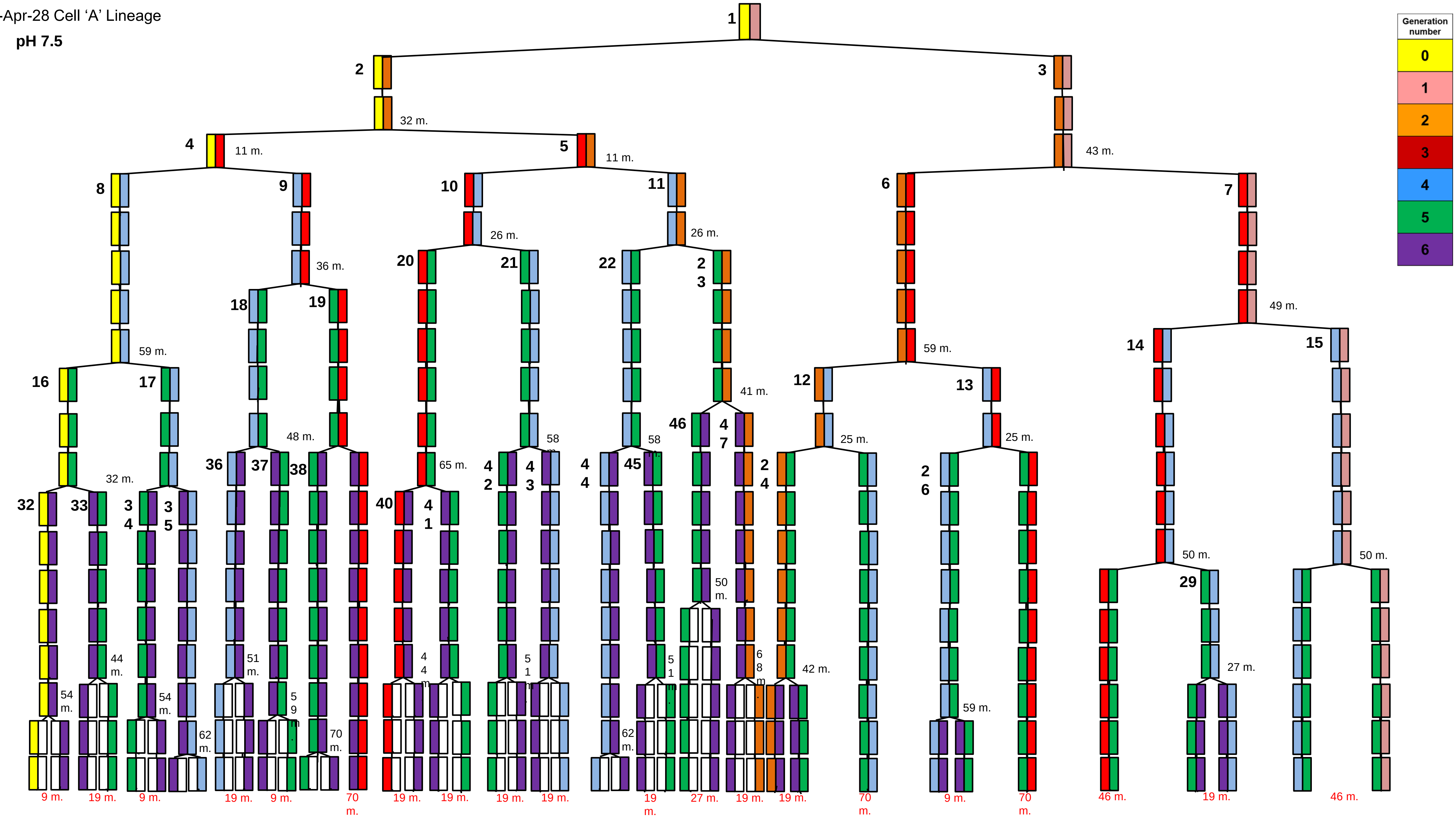

2014-May-29 Cell ‘B’ Lineage

pH 7.5

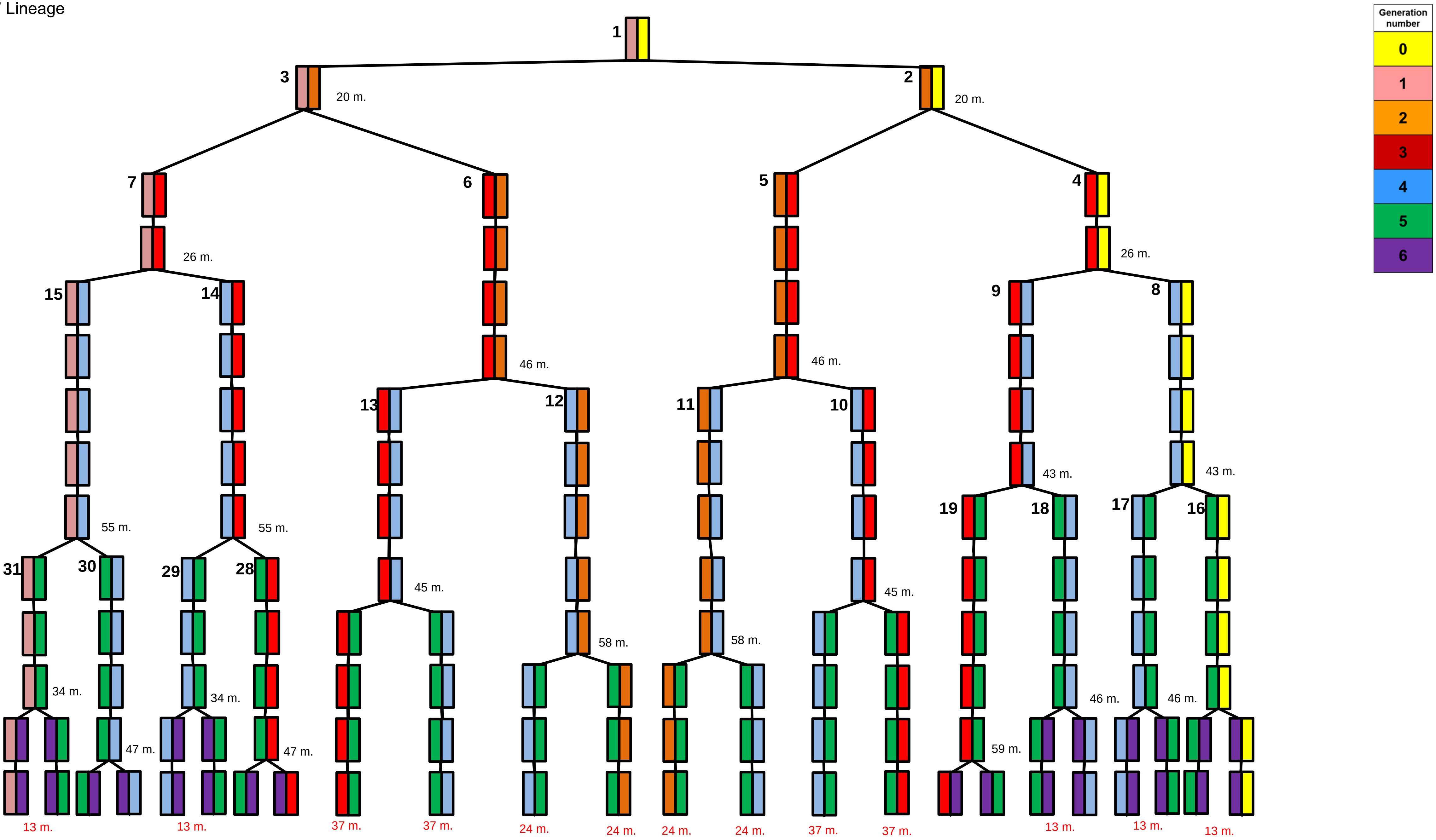

2014-July-17 Cell ‘A’ Lineage

pH 7.5

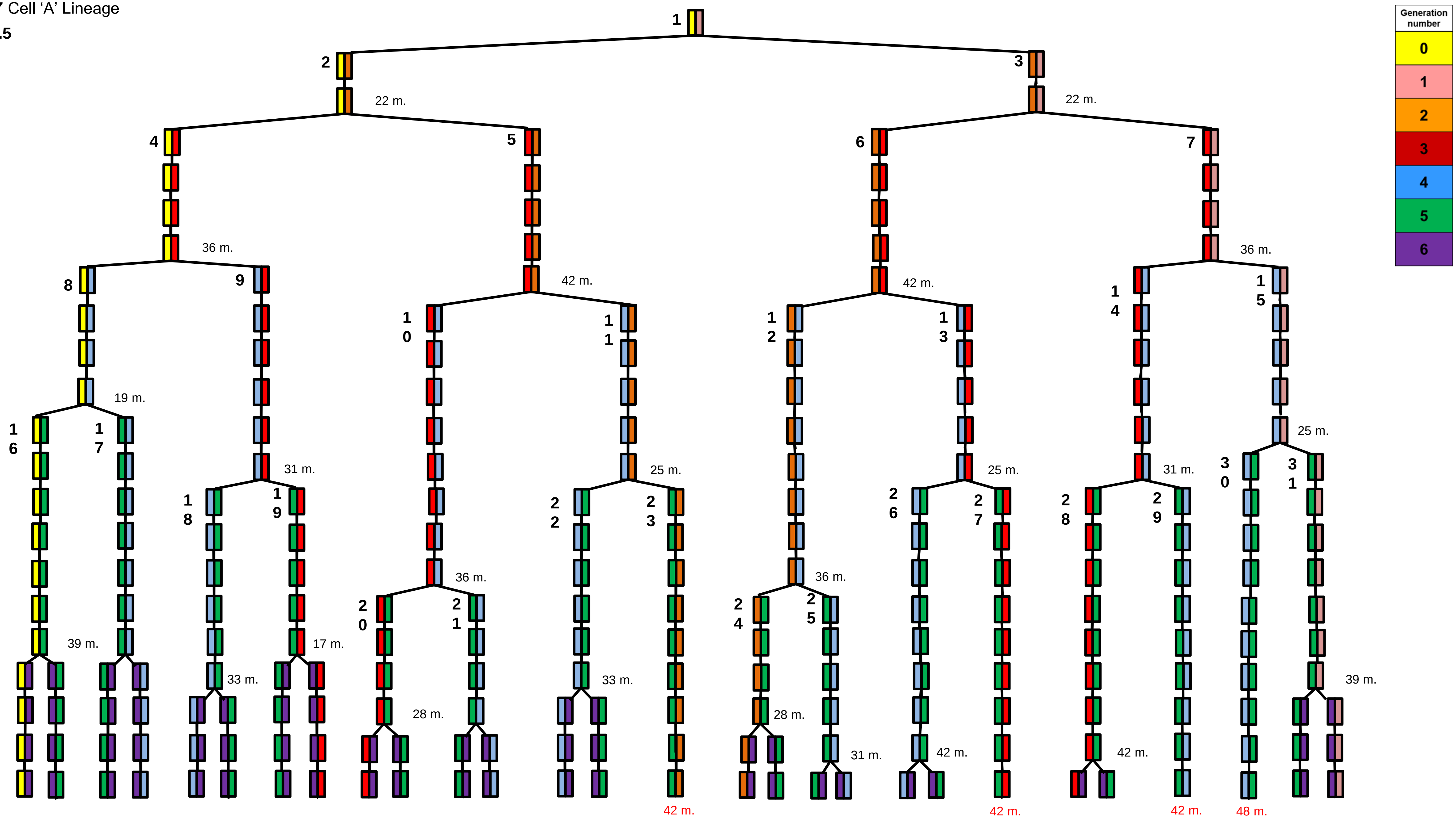

2014-July-17 Cell ‘B’ Lineage

pH 7.5

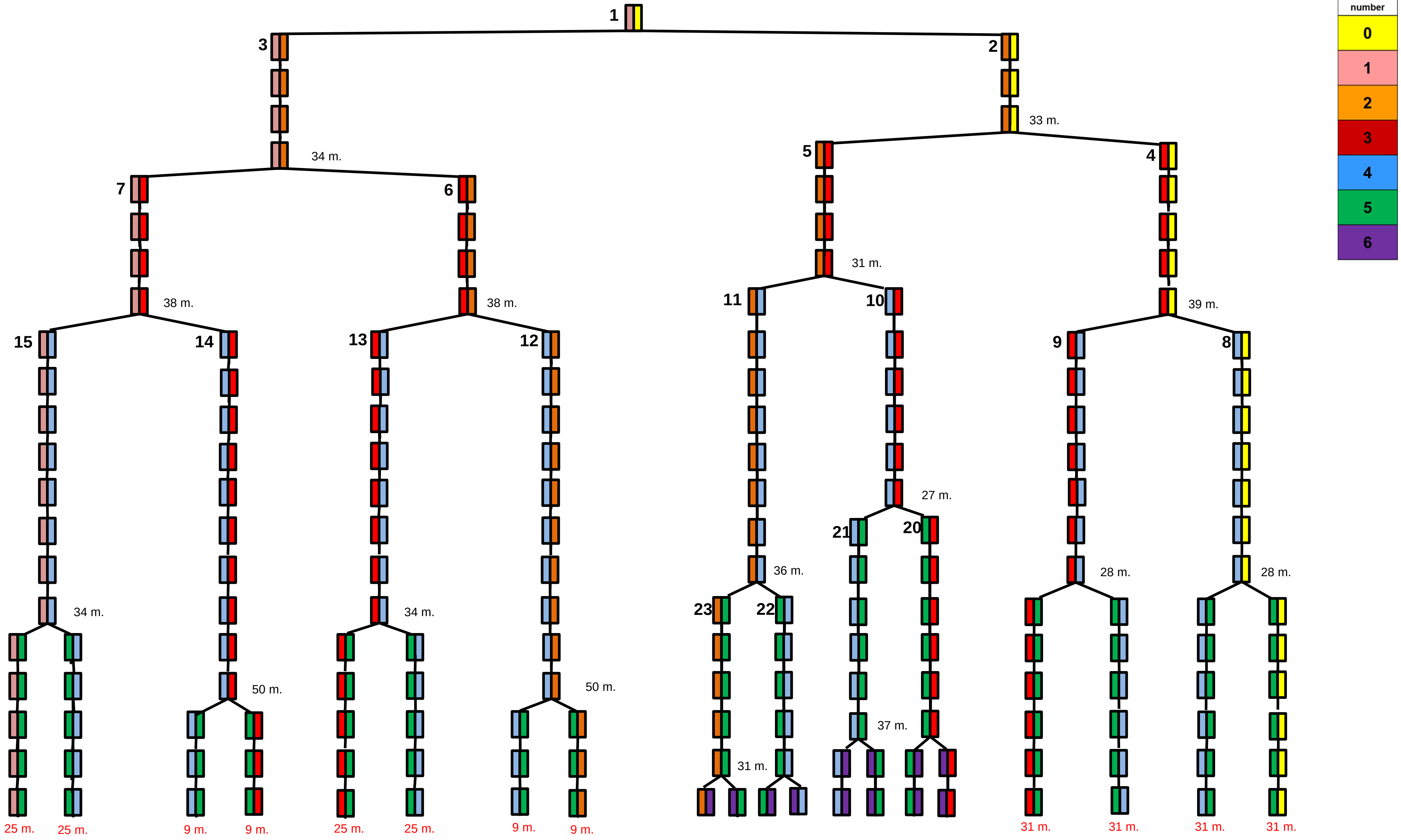

2015-Jan-6 Cell A

pH 7.5

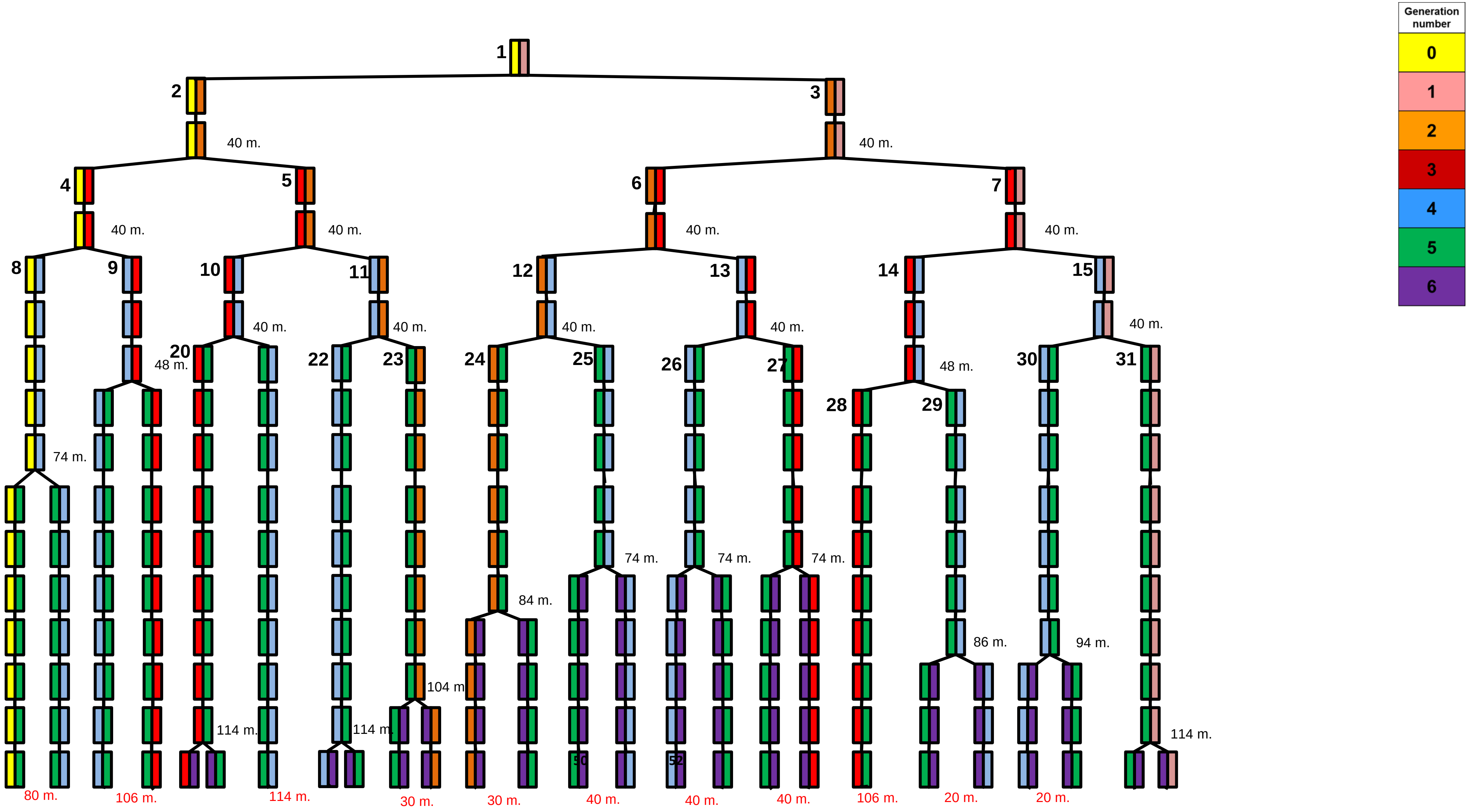

2015-Jan-15 Cell A

pH 7.5

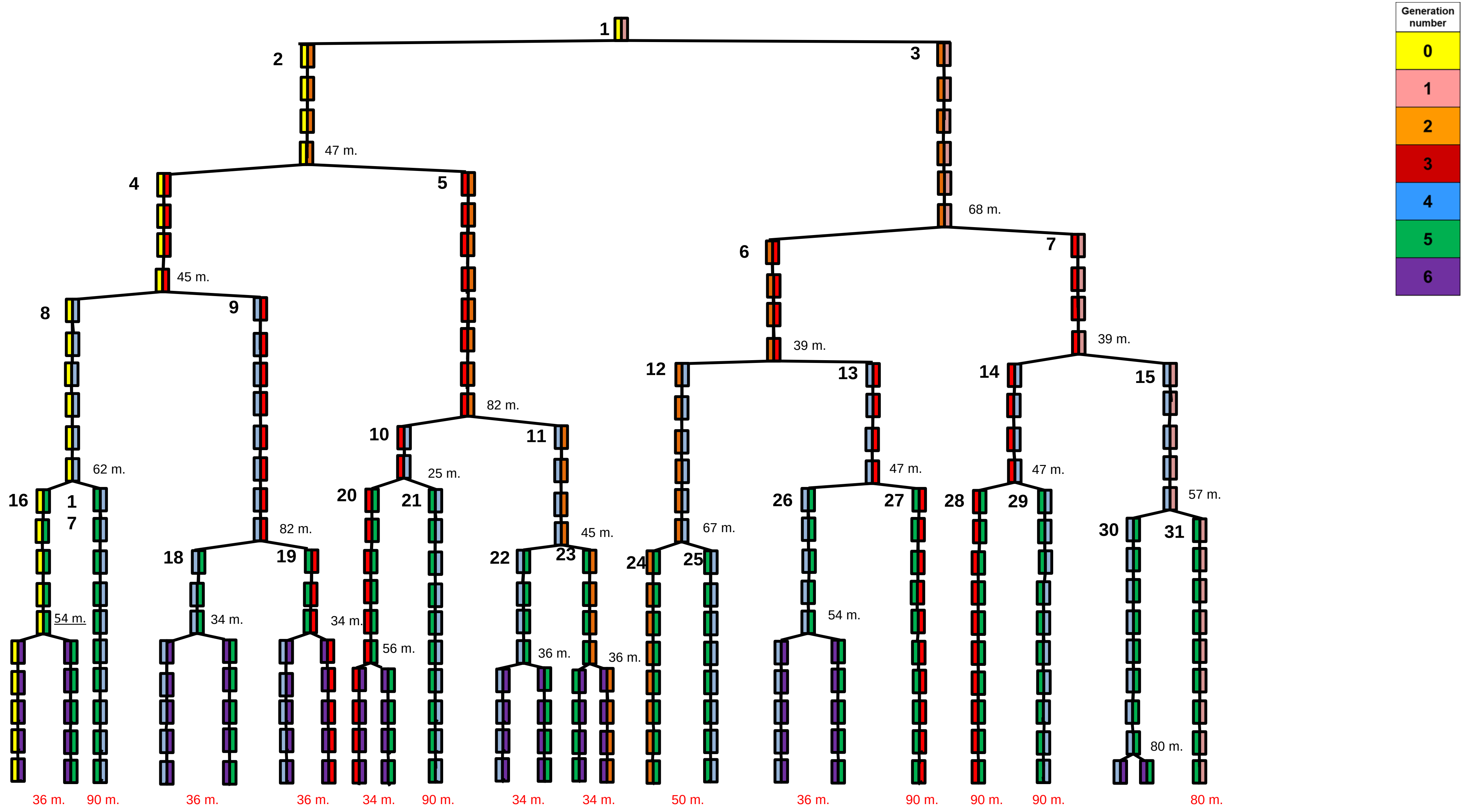

2015-Jan-15 Cell B

pH 7.5

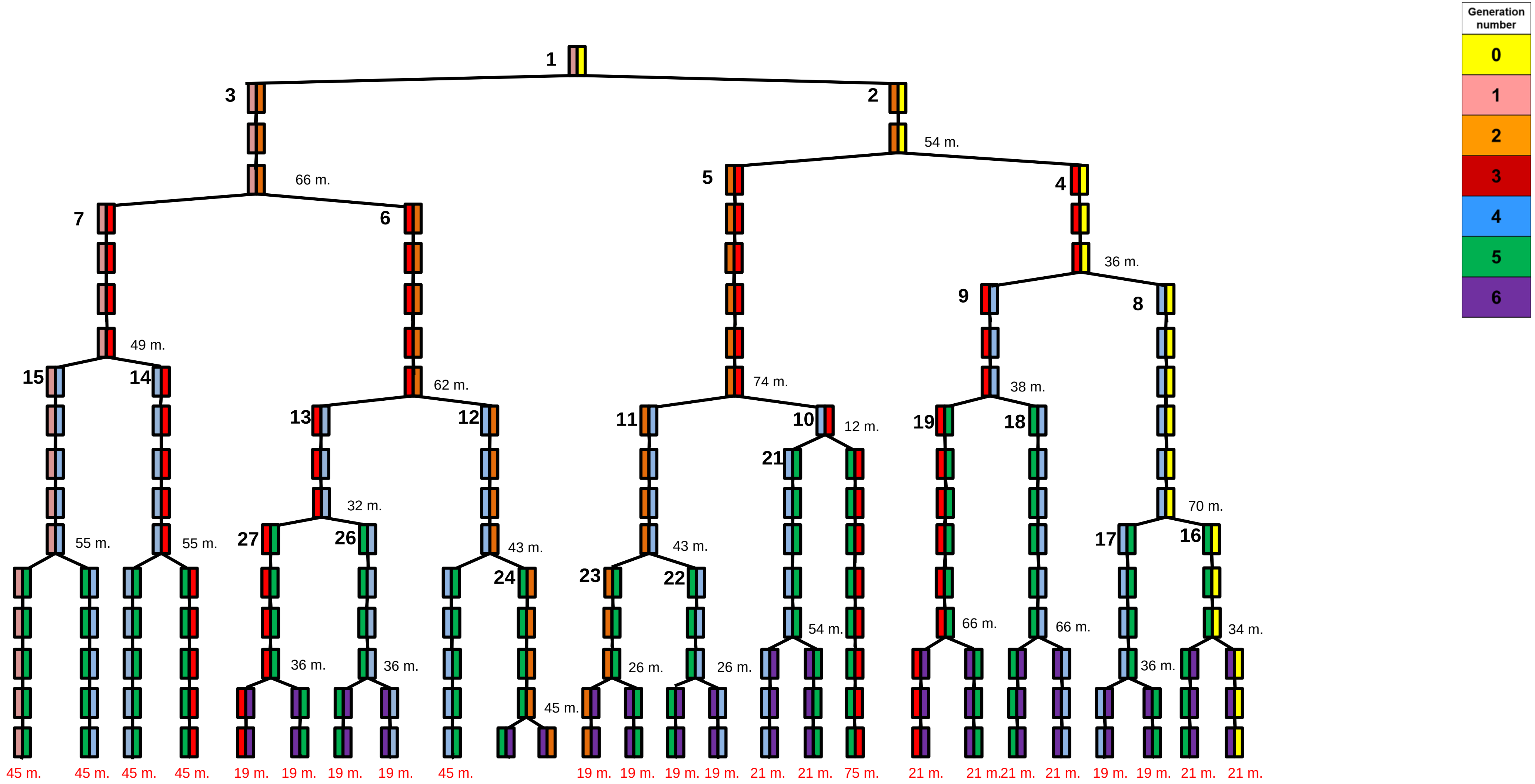

2015-Jan-22 Cell A

pH 7.5

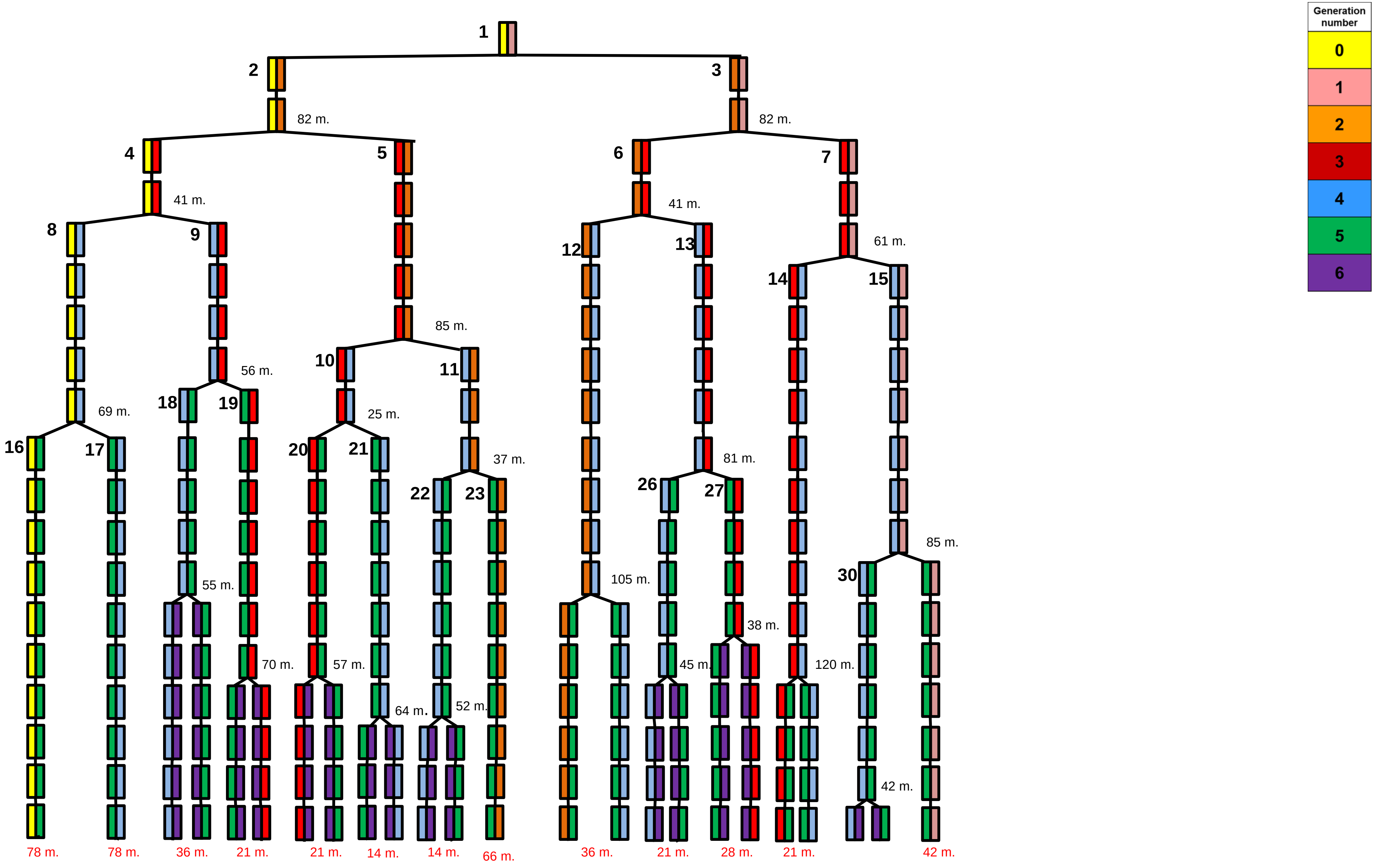

2015-Jan-22 Cell B

pH 7.5

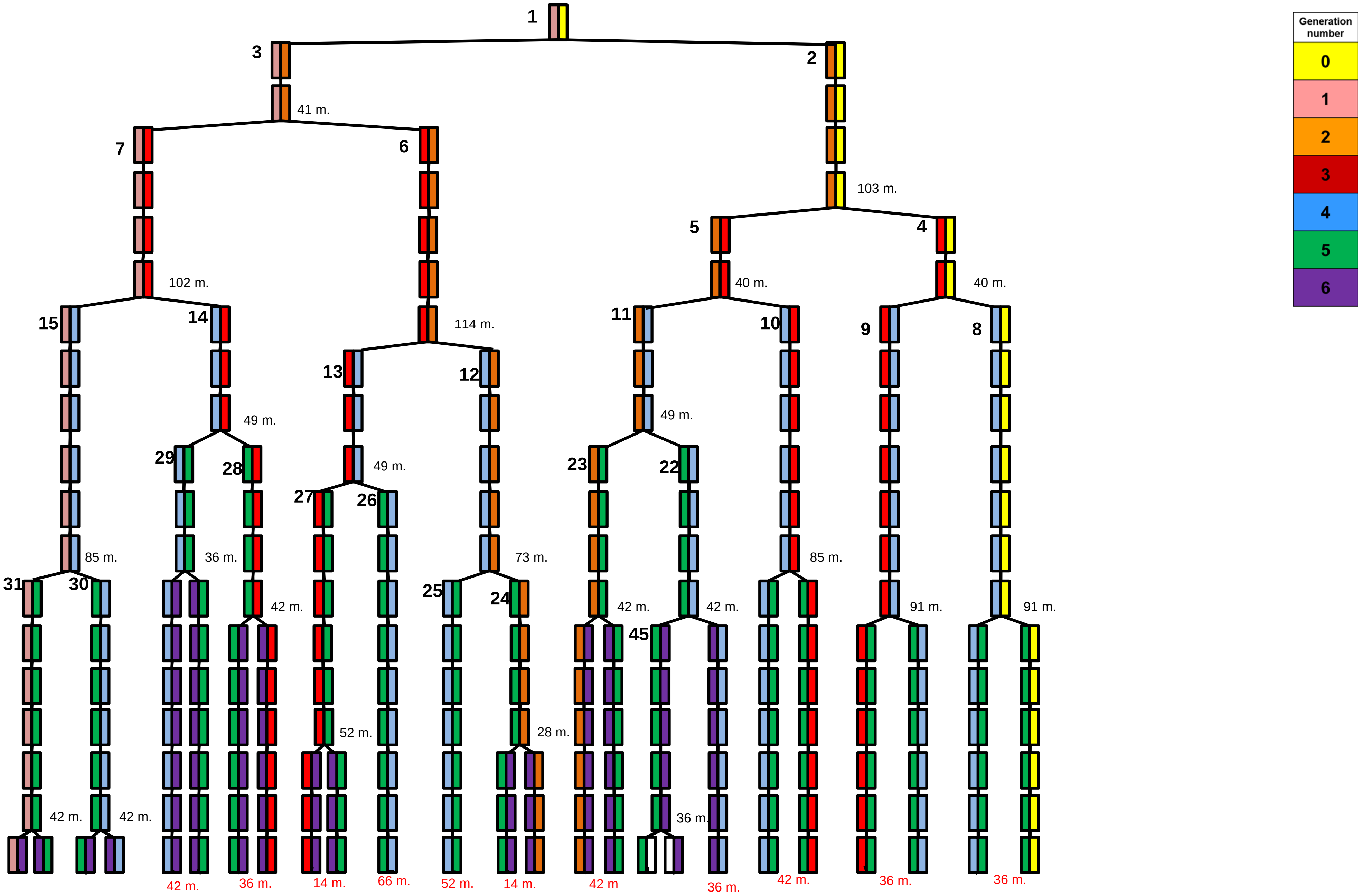

Supplement: S1 File — Fig A, Cell half-lineages of E. coli cultured in LBK buffered with 100 mM MES at pH 6.0. The lineages shown were included with those of Fig 2 for experimental analysis of the pH 6.0 condition. All lineages display at least five divisions for a total of at least six generations of cells in each experiment. Individual poles are color coded according to the color bar scale included in the upper right hand corner. Each cell in a half-lineage is also labeled with a distinct number next to the box. Cell numbers are standardized across all lineages. Time (min) at each box indicate the division time of that cell, the time from initial existence of the cell until the point where it divided into two daughter cells. Time (min) in red beneath the final division indicates the time the cell existed until the experiment was ended. Fig B, Cell half-lineages of E. coli cultured in LBK buffered with 100 mM MOPS at pH 7.5. The lineages shown were included with those of Fig 4 for experimental analysis of the pH 7.5 condition. Cells are labeled as for Figs A and B in S1 File. (PDF) [file pone.0144650.s001.pdf]
