# Supplementary material for: Periplasmic Acid Stress Increases Cell Division Asymmetry (Polar Aging) of Escherichia coli
Source: PLoS One. 2015 Dec 29;10(12):e0144650. doi: 10.1371/journal.pone.0144650 (PMC4694651; doi:10.1371/journal.pone.0144650)
Supplement: S2 File — Fig A, Intensity plot profiles of old-pole and new-pole cells at pH 6.0. Cells possessing the oldest pole (A) and the newest pole (B) were scanned end to end, from fluorescent images at 425 nm captured at the end of each experiment. Intensities were quantified using ImageJ (http://rsb.info.gov/ij/). Intensity scale is represented as low (0) to high (300). Lengths of cells are represented as pixels as indicates along the x-axis. Fig B, Intensity plot profiles of old-pole and new-pole cells at pH 7.5. Cells possessing the oldest pole (A) and the newest pole (B) were scanned end to end, and fluorescence was measured as for Fig A in S2 File. (PDF) [file pone.0144650.s002.pdf]

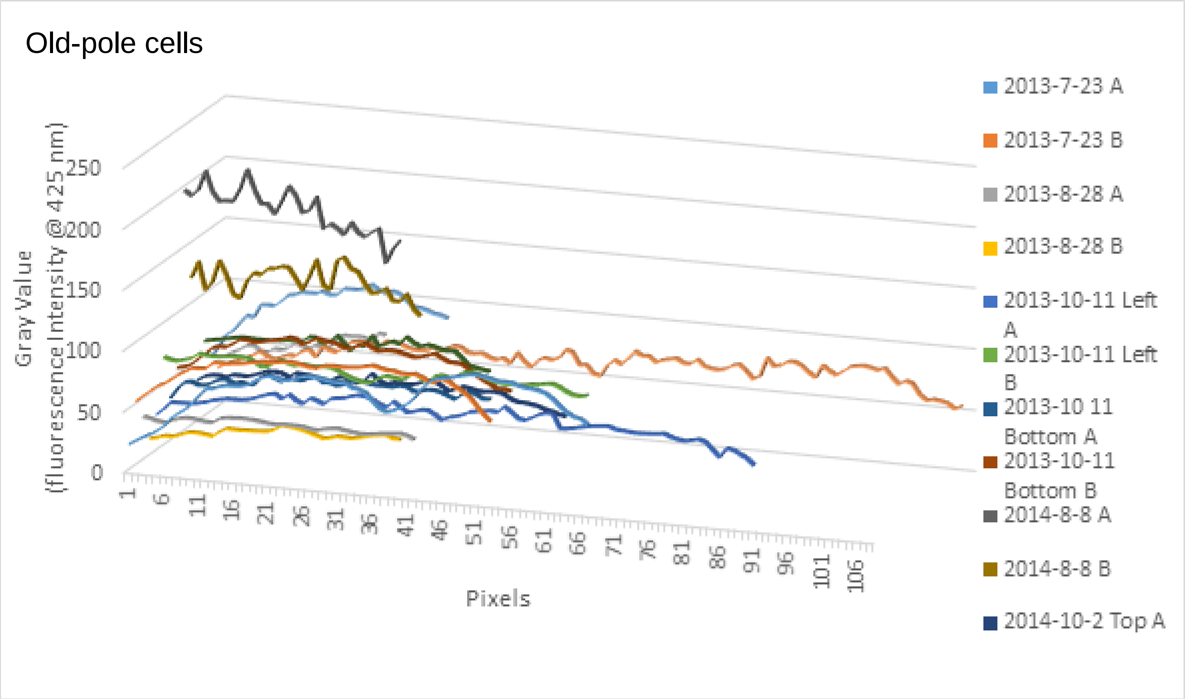

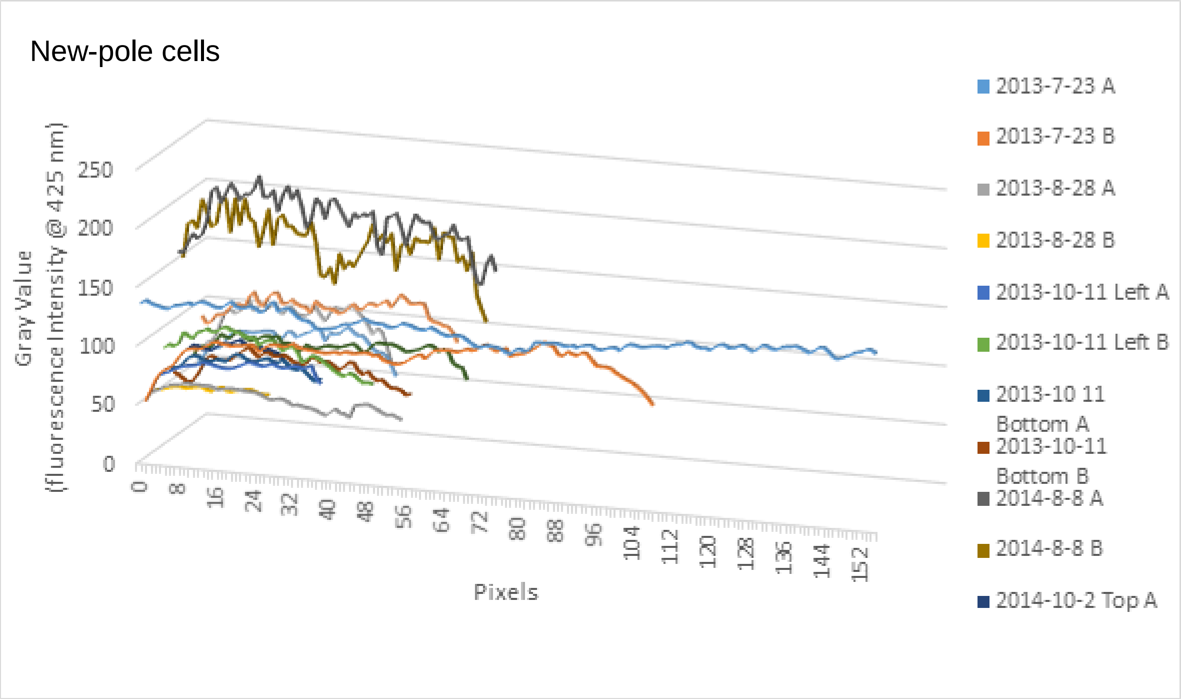

S2 Figure A. Intensity plot profiles of old-pole and new-pole cells at pH 6.0. Cells possessing the oldest pole (A) and the newest pole (B) were scanned end to end, from fluorescent images at 425 nm captured at the end of each experiment. Intensities were quantified using ImageJ (http://rsb.info.gov/ij/). Intensity scale is represented as low (0) to high (300). Lengths of cells are represented as pixels as indicates along the x-axis.

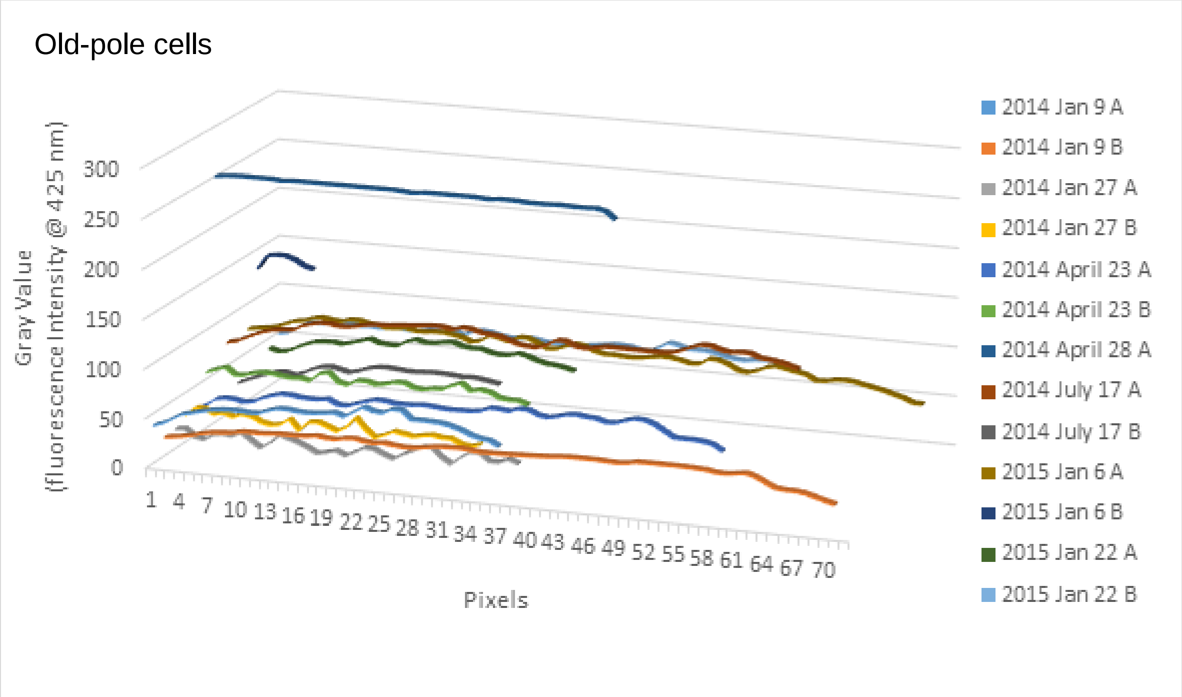

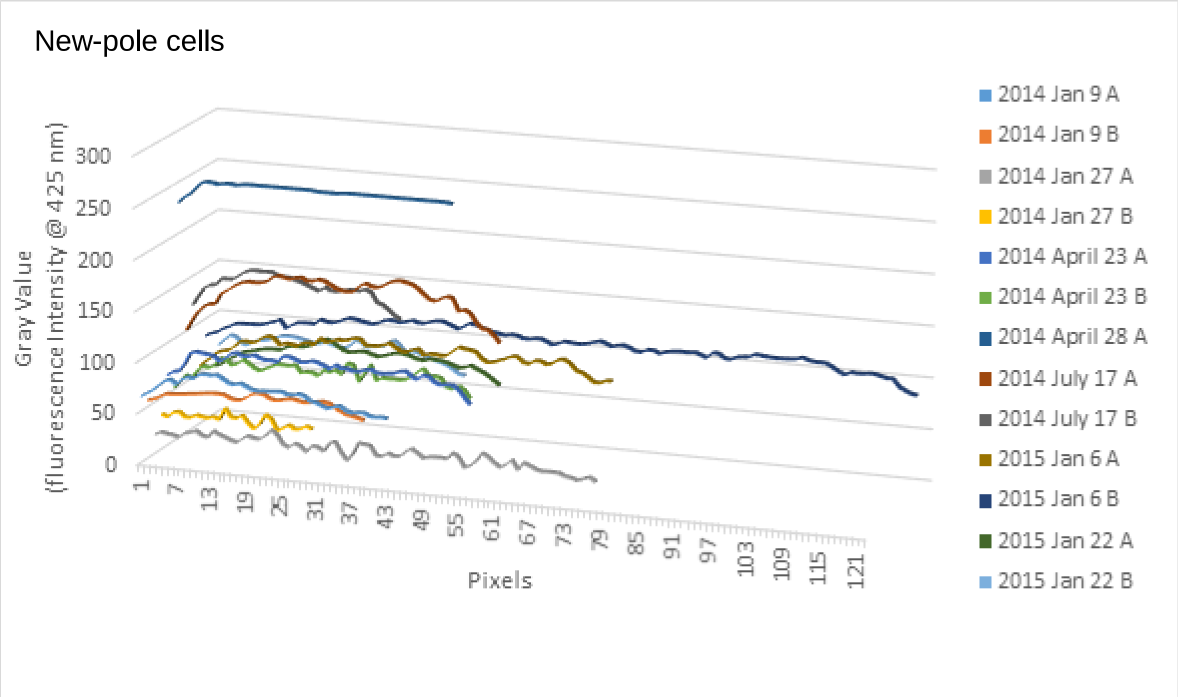

S2 Figure B. Intensity plot profiles of old-pole and new-pole cells at pH 7.5. Intensity plot profiles of old-pole and new-pole cells at pH 7.5. Cells possessing the oldest pole (A) and the newest pole (B) were scanned end to end, and fluorescence was measured as for Figure S3.
