# Supplementary material for: Periplasmic Acid Stress Increases Cell Division Asymmetry (Polar Aging) of Escherichia coli
Source: PLoS One. 2015 Dec 29;10(12):e0144650. doi: 10.1371/journal.pone.0144650 (PMC4694651; doi:10.1371/journal.pone.0144650)
Supplement: S3 File — Phase-contrast (top) and ratiometric fluorescence (bottom) images show the strain JLS1013, which expresses pHluorin under the constitutive promoter Pbsr [23]. Cultures were incubated at 37°C with rotation to stationary phase (14 h) in LBK media supplemented with 50 μg/ml ampicillin and buffered with 100 mM MOPS at pH 7.5. The cells were suspended in 0.35% agarose and spread on the 40 mm coverslip as described under Methods. The chamber was perfused with LBK media buffered at pH 7.5 (MOPS) during observation. Inclusion bodies resulted in regions of decreased fluorescence (arrow). (PDF) [file pone.0144650.s003.pdf]

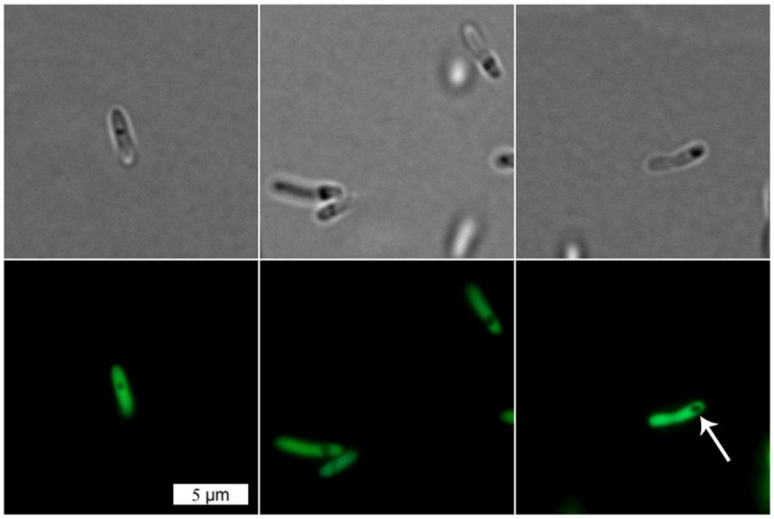

S3. Inclusion bodies observed in *E. coli* cells expressing pHluorin from P_{bsr}.

Phase-contrast (top) and ratiometric fluorescence (bottom) images show the strain JLS1013, which expresses pHluorin under the constitutive promoter P_{bsr} [23]. Cultures were incubated at 37 °C with rotation to stationary phase (14 h) in LBK media supplemented with 50 μg/ml ampicillin and buffered with 100 mM MOPS at pH 7.5. The cells were suspended in 0.35% agarose and spread on the 40 mm coverslip as described under Methods. The chamber was perfused with LBK media buffered at pH 7.5 (MOPS) during observation. Inclusion bodies resulted in regions of decreased fluorescence (arrow).
